# Molecular Characterization of *Ciborinia camelliae* Kohn Shows Intraspecific Variability and Suggests Transcontinental Movement of the Pathogen

**DOI:** 10.3390/microorganisms11112727

**Published:** 2023-11-08

**Authors:** Marco Saracchi, Irene Valenti, Paolo Cortesi, Daniela Bulgari, Andrea Kunova, Matias Pasquali

**Affiliations:** Department of Food, Environmental, and Nutritional Sciences, University of Milan, 20133 Milan, Italy; marco.saracchi@unimi.it (M.S.); irene.valenti@unimi.it (I.V.); paolo.cortesi@unimi.it (P.C.); andrea.kunova@unimi.it (A.K.); matias.pasquali@unimi.it (M.P.)

**Keywords:** camellia flower blight, morphological traits, UP-PCR, fungal diversity, *Sclerotiniaceae*, pathogen transmission, multi locus sequence typing

## Abstract

*Ciborinia camelliae* Kohn is the causal agent of camellia flower blight. The fungus infects only the flowers of camellias. *C. camelliae* isolates obtained from symptomatic samples, collected in 13 different localities worldwide, were characterized by Multi-Locus Sequence Typing (MLST) using the following: (i) a nuclear ribosomal DNA internal transcribed spacer; (ii) subunit 2 of β-tubulin (β-TUB II), (iii) elongation factor 1-α (EF1α); and (iv) glycerol-3-phosphate dehydrogenase (GPDH). The variability of the strains was assessed using a universally primed–polymerase chain reaction (UP-PCR) with six universal primers. Gene sequence comparison showed high similarity among all the European strains and highlighted the diversity of the New Zealand and Chinese representative strains. The profiles obtained by UP-PCR confirmed the significant diversity of extra-European strains and identified subgroups within the European population. The presence of shared genetic profiles obtained from strains isolated in different countries (New Zealand and France) suggests the movement of strains from one location to another, which is probably due to the exchange of infected plant material. Moreover, our study shows the overall high intraspecific variability of *C. camelliae*, which is likely due to the sexual reproduction of the fungus, suggesting the risk of emergence of new pathotypes adapting to novel camellia varieties.

## 1. Introduction

The ascomycete *Ciborinia camelliae* Kohn, inoperculate Discomycete of the *Sclerotiniaceae*, is the causal agent of camellia flower blight (CFB). It is a necrotrophic pathogen and shows host and organ specificity, infecting only flowers of species belonging to the genus *Camellia*, causing serious damage to the ornamental component of the plant [1,2].

The pathogen was first reported in Japan in 1919 [3,4] and is now present in several countries worldwide where camellia plants are cultivated. According to the European and Mediterranean Plant Protection Organization (EPPO) database, the fungus is present in Europe (France, Germany, England, Ireland, Italy, Netherlands, Portugal, Spain and Switzerland), Japan, New Zealand and North America (California, Florida, Georgia, Louisiana, South and North Carolina, Texas and Oregon). Regarding the diffusion of the pathogen in Italy, our previous studies confirmed the presence of CFB in Piedmont, Tuscany, and Lazio, and we reported its occurrence in Emilia Romagna, Lombardy, Liguria, and Campania for the first time [5]. Nevertheless, the distribution data are neither detailed nor updated. 

Characterizing the population variability is a useful tool to investigate the pathogen ecology, trace its evolution and understand the movements of the pathogen among different territories. Furthermore, a plant pathogen’s phenotypic characterization could help disease management [6,7,8,9].

In the literature, information about *C. camelliae* is lacking, especially concerning pathogen populations and their variability. The diversity of the fungus has been investigated by exploring random-based genetic approaches (e.g., UP-PCR) as well as sequence-based approaches on a group of New Zealand and American strains, demonstrating that the level of genetic variation within the two populations was relatively low [10,11,12]. The latter was based on ITS sequence diversity alone, but recent works in the fungal domain have demonstrated the numerous advantages of Multi-Locus Sequence Typing to decipher fungal diversity and evolution [13]. The phenotypic variability of numerous strains isolated from phytopathological samples collected in different Italian provinces has been described in a recent paper by Saracchi et al. (2022) [2], demonstrating a significant variability among the isolates obtained from the same country. 

To ascertain the variability of the pathogenic fungus *C. camelliae*, the level of genetic diversity was assessed by considering strains of different origins and using different molecular markers. Nuclear ribosomal DNA internal transcribed spacer (ITS) and genes coding for subunit 2 of β-tubulin (β-TUB II), elongation factor 1-α (EF1α) and glycerol-3-phosphate dehydrogenase (GPDH) are frequently considered for fungal identification at the genus and species level and to explain the evolutionary relationships among species [14,15,16,17]. These molecular markers have also been used in various studies to highlight intraspecific variability [18,19,20,21,22,23,24,25,26,27,28]. For these reasons, one of the aims of this study was to evaluate the genetic variability amongst 47 isolates of *C. camelliae* from 13 worldwide localities by comparing the nucleotide sequences related to these regions.

Subsequently, to investigate in detail the variability of the studied population, universally primed–polymerase chain reaction (UP-PCR) markers were used. This technique combines high annealing temperatures and universal primers [29] targeting different regions of the genome with polymorphic features, which is useful to determine genetic diversity also among isolates belonging to the same species [30]. 

The construction of phylogenetic trees and the cluster analysis of the data highlighted differences among strains isolated from different countries, in particular between the isolates of Chinese and New Zealand origin and those from Europe, and indicated the recent movement of strains between continents.

## 2. Materials and Methods

### 2.1. Fungal Isolation and Morphotypes

Symptomatic flowers and sclerotia associated with *C. camelliae* infections were collected in 7 different localities worldwide from 2016 to 2021. Strains and sclerotia obtained from New Zealand diseased camellias have been also included: strain C5.19 was kindly provided by N. Kondratev (Palmerston North), and two strains isolated from sclerotia were sent by R.F. van Toor (Lincoln, Canterbury, UK).

Pathogenic strains were isolated on potato dextrose agar (PDA: 800 mg/L of potato extract; 20 g/L glucose, BioFROXX, Germany; 15 g/L agar, Applichem, Darmstadt, Germany) and PDA added with antibiotics (PDA+++; nalidixic acid 25 mg/L, Sigma-Aldrich, Darmstadt, Germany; tetracycline 25 mg/L, Applichem, Darmstadt, Germany; novobiocin 25 mg/L, Sigma-Aldrich, Darmstadt, Germany) to prevent bacterial growth. Untreated infected flower tissues or sclerotia were sterilized by soaking for 3 min in a solution (50/50 *v*/*v*) of 95% ethanol and 5% NaOCl and washed three times in sterile distilled water. Flowers were cut into small pieces and placed onto culture media. Colonies of the pathogen were selected based on (i) colony morphology and (ii) a microscopic investigation of mycelium and conidia structures. 

The cultural and morphological characterization of 25 strains and their morphotype pattern were assessed as previously described [2]. 

Twenty-two strains previously isolated from phytopathological samples collected in Italy representing all the morphotype patterns found in each of the six different localities [2] were also included to this study. 

Infection assays to prove Koch’s postulates were carried out on three representative *C. camelliae* strains from three continents: ITAC2 (Europe), C5.19 (New Zealand), and PRC26 (Asia). Newly bloomed flowers, present on branches cut from plants belonging to the “Sacco”, and “Apple Blossom” varieties and a *C. japonica* × *C. azalea* hybrid, were inoculated experimentally by placing small drops of aqueous suspensions containing fragments of fungal mycelium (1000 CFU/mL) on the petals. Flowers treated with sterile deionized water were used as negative control. The flowers were kept for 24 h in a confined environment at 20 °C and 90% RH; then, the latter was lowered to 60%. The flowers were observed periodically to check for the development of symptoms consisting of necrotic areas and finally detected after 7 days. All strains used in the present study are listed in Table S2. They are maintained in the culture collection of the Laboratory of Plant Pathology at the Department of Food, Environmental and Nutritional Sciences (DeFENS), University of Milan, Italy.

### 2.2. *Ciborinia camelliae* Mycelia Production and DNA Extraction

Pathogen agar–mycelium plugs taken from the edge of a colony actively grown on PDA medium were placed in potato dextrose broth (PDB, Difco, Detroit, MI, USA). All flask cultures were incubated at 20 °C for 5 days with shaking at 125 rpm. After the incubation, colonies were transferred to sterile Falcon tubes, centrifuged, and washed twice with sterile distilled water to remove traces of the medium. Mycelium was lyophilized (model Heto-EPD3; Thermo Scientific, San Jose, CA, USA) for 24 h and then ground with fine sterile sand in a mortar. Finally, the mycelium powder was stored at −20 °C.

DNA was extracted and quantified according to van Toor et al., 2005 [11]. 

### 2.3. PCR Amplification

Genetic diversity was assessed by MLST comparing the sequences of specific DNA: (i) a nuclear ribosomal DNA internal transcribed spacer (ITS) and genes coding for (ii) subunit 2 of β-tubulin (β-TUB II), (iii) elongation factor 1-α (EF1α) and (iv) glycerol-3-phosphate dehydrogenase (GPDH) (Table 1) and by universally primed–polymerase chain reaction (UP-PCR) [11] (Table S1). For elongation factor 1-α and GPDH amplification, new primers were designed using Primer3 web software, version 4.1.0 [31] upon identification of the two genes in the raw genome of the strain ICMP 19812 (Genbank: LGQK01001007) from New Zealand (Massey University Arboretum, Palmerston North). 

All PCR products were amplified using GoTaq^®^ DNA Polymerase (Promega, Madison, WI, USA) in a total volume ranging from 25 to 30 microliters using Gene Cycler (BIO-RAD laboratories, Segrate, Italy).

The UP-PCR was performed according to van Toor and coworkers (2005) [11]. All PCR products were resolved in 1.5% agarose gel electrophoresis containing ethidium bromide, visualized using UV-transilluminator Gel Doc 2000 (BIO-RAD laboratories, Hercules, CA, USA) and analyzed by Quantity One software v.4.0 (BIO-RAD laboratories, Segrate, Italy.

### 2.4. Gene Sequencing, Phylogenetic Analyses and UP-PCR Banding Profiles

The PCR products of gene amplifications were sequenced by Eurofins Genomics (Ebersberg, Germany) using the Sanger technology, and all sequences were verified, aligned, assembled, and trimmed using Geneious software (Biomatters, Auckland, New Zealand) version R11.1.4 using the same primers.

For the phylogenetic analyses, the four gene sequences of each strain were considered both separately and concatenated. Relationships among strains were carried out using IQ-TREE server version 2.2.2.6, which is a phylogenetic software for maximum likelihood analysis [34]. The graphical tree representation was developed by Molecular Evolutionary Genetics Analysis (MEGAX) software version 11.0 [35] using the NEWICK format of the maximum likelihood tree. 

For each combination isolate/universal primer, the bands were scored only if they were clearly detectable under the UV light. Matrices based on the fragment absence (0) or presence (1), both in relation to each single primer and to all six considered as a whole, were arranged. Following van Toor and coworkers [11], pair-wise similarities were calculated between strains using Jaccard’s coefficient of similarity *Sj* = *a/*(*n* − *d*), where *a* represents the number of 1–1 matches, *d* represents the number of 0–0 matches and *n* represents the total number of bands compared. To compare the genetic similarity among strains, dendrograms were constructed according to the UPGMA algorithm using NTSYS pc v2.2 [36], as mentioned previously.

Differences between known groupings based on geographical origin were assessed by canonical variate analysis [37], which compared the within-group variances and covariances of the bands with the between-group variances and covariances describing the patterns of diversities among the locations [10].

## 3. Results

**Figure 1:**
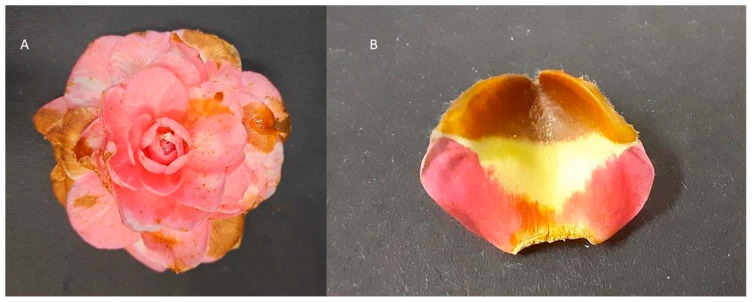
*Camellia japonica* “Sacco” symptomatic flower (**A**) and petal (**B**) from experimental inoculation using *C. camelliae* strain ITAC2.

The 47 strains of *C. camelliae* isolates from 13 locations from Europe, New Zealand and China were classified in 16 morphotype patterns (Table S2). Twenty-two strains representing eleven morphotypes were described previously [2], while the morphotypes of 25 strains were determined in this study. Based on the morphology of the colonies of the 25 non-Italian strains grown on four different media, it emerged that eight strains (CH14, CH16, NT1, PT1, PT4, PT7, PT8, SPA4) showed a unique combination of colony morphologies, resulting in five new morphotype patterns, with the pattern 16 being the most prevalent (Table S3). Infection with the three representative strains confirmed that from the fourth day of incubation, brownish necrotic areas developed on all flowers of three varieties inoculated with all three strains. The maximum size of the lesion occurred 7 days after inoculation. Fungi referable to *C. camelliae* were reisolated from the necrotic tissues, fulfilling Koch’s postulates (Figure 1).

### 3.1. Gene Sequencing and Phylogenetic Analyses

**Figure 2:**
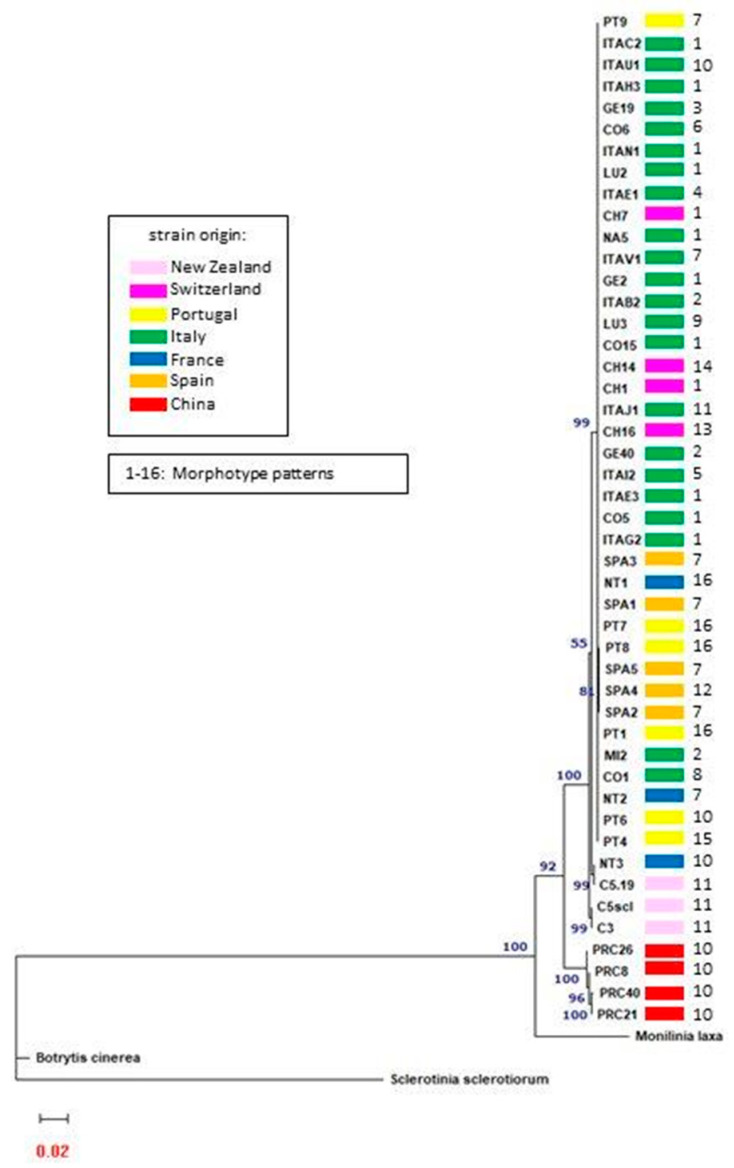
The phylogenetic tree calculated on the concatenated ITS, β-TUB II, EF1α, and GPDH nucleotide sequences of the 47 *C. camelliae* strains and three different species belonging to *Sclerotiniaceae* family as outgroups, using the model K2P + R3 selected by the IQ-TREE server version 2.2.2.6. Numbers above the nodes indicate bootstrap support values. Numbers next to the strain identifiers indicate morphotypes.

Sequences of the 47 strains for the ITS, β-TUB II, EF1α, and GPDH regions were deposited in Genebank (Table S3). Phylogenetic trees for each target were mutually concordant (Figures S1–S4). Based on this result, the sequences were concatenated to form a single sequence of 1867 nucleotides for each strain. The phylogenetic tree constructed from the concatenated gene alignments shows that all *C. camelliae* strains group into a single group that clearly differs from the outgroups (Figure 2). *Botrytis cinerea* is the most dissimilar from *C. camelliae*, while *Monilinia* has sequences that most resemble those of the pathogen. *Sclerotinia sclerotiorum* differs from *C. camelliae* as a separate species, showing intermediate dissimilarities among the considered outgroups.

The four Chinese strains form a single group that is separated from all the other *C. camelliae* strains (Figure 2). The ITAC2 isolate, whose draft genome sequence was recently published [38], was considered the representative strain of the most numerous group characterized by identical sequences. The Chinese strains show 96.4% similarity (100 mismatches and 4 gaps) with the ITAC2 strain.

Two of the three New Zealand strains (C5scl and C3) differed from the main group of the 43 remaining strains in 23 mismatches and 1 gap from the ITAC2 strain (99.2% similarity). Both strains were isolated from sclerotia collected in Lincoln (NZ). The third New Zealand strain (C5.19), coming from camellias grown in Palmerston more than 200 km further south, showed 99.4% similarity to the majority of the investigated strains, and its concatenated sequence differed by only 18 mismatches. Interestingly, the French strain NT3 collected in Nantes (France) was identical to the New Zealand C5.19 strain.

The 1867 nucleotide sequences of the 41 remaining strains exhibit 100% similarity to each other. Therefore, most of the examined strains form a single group.

### 3.2. Universally Primed-PCR Analysis

To better assess the relationships among the strains, the band profiles related to each strain were evaluated by analyzing the electrophoresis gels of UP-PCR products. The six primers produced from 15 (AA2M2) to 34 (As15inv) polymorphic bands, whose size ranged from 140 to 3500 bp (Table 2). 

**Figure 3:**
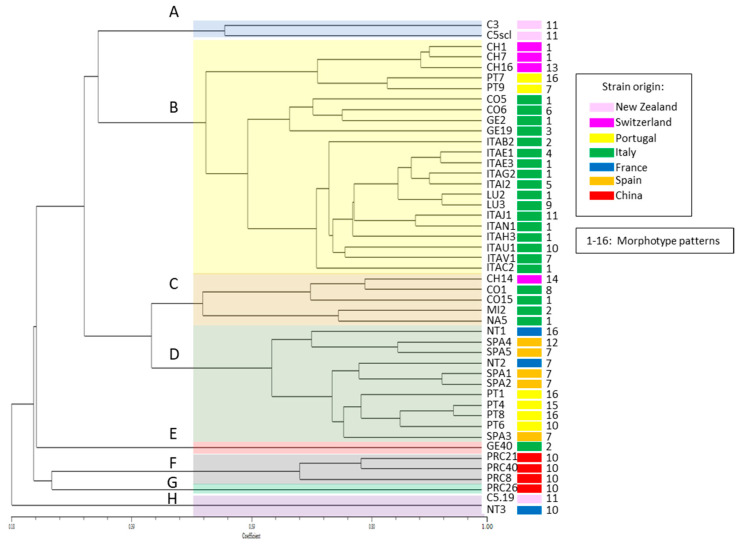
Similarity tree of the genetic relatedness of *Ciborinia camelliae* strains based on UP-PCR fragment analysis calculated on Jaccard’s coefficient and the UPGMA cluster method. Colored areas indicate groups (**A**–**H**) of similar strains according to similarity coefficient (>0.5), and the color next to each strain indicates the geographic origin of the strains. Numbers next to the strain identifiers indicate morphotypes.

Based on the presence/absence of each polymorphic band (144 in total, Table S5, Figure S5), similarities between all the possible pairs of strains were calculated, and the hierarchical cluster analysis based on Jaccard’s coefficient was produced (Figure 3). In total, eight separate clusters (A–H) can be distinguished based on the similarity coefficient (>0.5). Only 2 strains out of 47 showed the same profile: C5.19 from New Zealand sclerotia and NT3 from Nantes, which also formed a separate group based on the concatenated nucleotide sequence of four DNA regions (Figure 2), confirming the high level of similarity between two distantly collected strains (cluster H).

This variability study highlighted that also the Chinese strains greatly differ from all the other strains (clusters F and G). Even though the within-group variability among the Chinese strains can reach 75%, they form a group that differs by a further 3.8% from all the other members of the tree.

The strain GE40 (cluster E) showed a significant propensity to differentiate from the average profile of the main group of analyzed strains. This group is divided into two numerically similar groups, which share 30.7% of the UP-PCR profile.

Grouping can be partially explained by the geographical origin of the strains. Some strains with common origin show a very similar profile to each other, but they often differ significantly from others with similar origins.

Two of the New Zealand strains (C3 and C5scl, cluster A), both from samples collected in the same locality, still show significant differences (70% similarity coefficient) from the average profile of the larger group of strains.

As can be seen in Figure 3, excluding the small groups that already differed greatly from the remaining strains (four from China, one from New Zealand, France and Italy), the bulk of the population is divided into four groups (A–D).

The largest group is cluster B, which includes most of the Italian strains isolated from samples collected in four different Italian provinces (Verbania, Como, Genoa, and Lucca) and a more limited subgroup with three Swiss and two Portuguese strains. The percentage of variability among the 22 strains is 49.23%.

Groups C and D are the most heterogeneous from the geographical point of view and include Italian, Swiss, Spanish, French and Portuguese strains. The strains belonging to group C are more variable than those in group D, the intra-group variability is, respectively, 49.9 and 38%.

**Figure 4:**
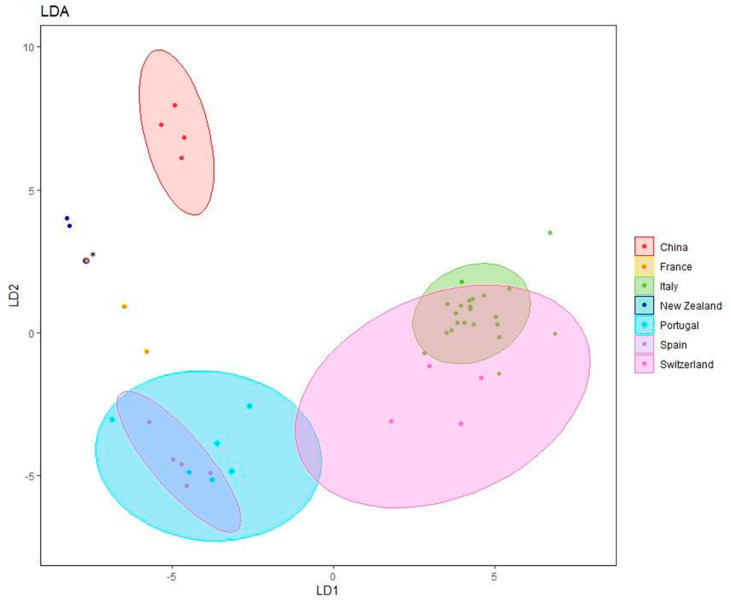
Canonical variate analysis plot of UP-PCR bands. * Overlap between NT3 and strains C5.19 coming, respectively, from France and New Zealand.

From the analysis of the canonical variables, which are summarized in Figure 4, the tendency of the strains to group themselves by geographical origin can be observed. The similarity among strains from Italy and Switzerland and those from Spain and Portugal seems quite consolidated. Equally, the diversity of the New Zealand and Chinese strains from those of Europe appears evident. This analytical approach confirmed the diversity among the numerous strains isolated in Italy in different areas that are sometimes geographically distant.

## 4. Discussion

Over the years, many aspects of *C. camelliae* taxonomy and its relations with the family *Sclerotiniaceae* have been clarified [4,39,40]. On the other hand, the information on its ecology, the relationships with both environmental factors and the host, and the uniformity of its population are still limited [1,41]. A high percentage of publications report only the presence of the disease, as reviewed by Taylor and Long [1] and Saracchi et al. [41]. We collected 220 strains from different continents and countries, describing morpho-cultural characteristics and confirming their taxonomical identification at the molecular level. In this study, five additional morpho-cultural profiles were detected, suggesting a high level of polymorphisms of the fungus. The use of the MLST analysis with the nuclear ribosomal DNA internal transcribed spacer (ITS) and three nuclear genes (β-TUB II, EF1α, and GPDH), which showed effective discriminatory ability in the fungal kingdom [42], indicated an unexpectedly high level of intraspecific variability between the New Zealand and Chinese strains compared to the European ones, which on the contrary showed a high level of similarity. The relatively high diversity of the Chinese group suggests the possible existence of a diverging evolutionary pathway that might lead to a speciation phenomenon [13].

The comparison of the UP-PCR profiles confirmed the significant diversity of the extra-European strains and highlighted the variability among the European ones. Since the group of Italian strains is particularly large, the study allowed detecting variability both among strains coming from distant locations as well as from the same geographic site. Previous amplification profiles using UP-PCR [43] by van Toor and colleagues were partially different from our profiles. This is likely due to the different timing of samplings: the strains used by van Toor were isolated in 2001, while those used in this study were collected at least 15 years later. Given that the pathogen is characterized by an elevated potential genetic variability due to sexual reproduction [1,41], this could have contributed to a significant genetic modification of the populations. Considering both studies, the physical separation of the pathogen in the two different geographic areas may have contributed to enhancing the differences. In New Zealand, the pathogen was first reported in 1993–1994, while in Europe, it was first reported in the early 2000s. In both cases, the analyses of the population variability were carried out about fifteen years later. In van Toor’s work, low variability is reported among strains of the same geographical area.

As far as European countries are concerned, the high morphological and/or genetic differences of *C. camelliae* strains may have been influenced by several factors. Indeed, many morphotypes are present in different countries, while some are mostly associated with specific geographic regions (Figure 3 and Figure 4). The temporal diffusion of the disease into various countries is not certain. The disease could have appeared already before 2000, being not officially reported, as it may have started in private green areas whose owners were not aware of the need to report the presence of a new disease or, in some cases, the reports were not made for fear of restrictions related to quarantine laws. The high variability of the populations at the continental level, not reported for New Zealand strains in the early 2000s [11], can be partially justified by the gene recombination due to sexual reproduction and by the spread of windborne ascospores, which can travel anywhere from several meters to several kilometers [44,45,46]. On the other hand, this diversification could also be partially, and involuntarily, increased by the movement of fungal propagules by collectors who frequently pick up camellia plants during their travels and bring them to their countries to enrich public or private collections. The importance of this latter aspect also depends on the number of people involved in the cultivation and collection of camellias and the ease of travel among geographical locations where the camellias are grown. Obviously, the exchange between European countries is easier than with distant ones such as New Zealand or China. Therefore, it is possible to find very dissimilar strains in the same sampling area and, at the same time, similar strains in areas distant from each other. Interestingly, the high similarity between French and New Zealand isolates observed by UP-PCR and MLST analysis may indirectly suggest the movement of infected camellias between the two regions confirming, as reported for many pathogens before, that pathogens can easily move with propagation materials [47,48,49]. This was further confirmed by the complete MLST sequence and UP-PCR profile identity of French and New Zealand isolates, as reported in Figure 4 [47,48,49]. Studies on the diversity within the *Sclerotiniacae* family showed that variability among strains can be very high [50], but it can also be very limited in space and time [51], suggesting the need to better decipher the different genetic backgrounds of *Sclerotiniaceae* undergoing fast genetic changes, or on the contrary, high genetic stability. 

However, such a distribution of similar strains does not allow us to demonstrate with certainty all the movements from one nation to another. Common origin can be hypothesized among the strains isolated from the material collected in the provinces of Verbania, Como, and Switzerland, which are areas close to each other. A similar event may have occurred for the strains from Spain and Portugal. Geographic isolation can also explain the uniformity of strains isolated from Chinese camellias, which are very different from all the other strains under study. 

Future work may consider expanding the number of strains investigated, including other geographical areas where the disease is present but from which it has not been possible to obtain new strains yet. It would also be appropriate to increase the number of genetic markers to better understand the relationships among strains, which would help reconstruct their origins and their possible movements among different geographical areas [52,53]. It would also be interesting to study the variability of the *C. camelliae* population in relation to (i) the sensitivity of camellia varieties to the disease, (ii) the relationships between the host and the pathogen, and (iii) its possible influence on disease control with both agronomic methods and biocontrol agents. This work represents the first comprehensive study on the *C. camelliae* molecular variability of isolates from three continents.

## Figures and Tables

**Table 1:** Primers and corresponding amplification targets and annealing temperatures. References concerning developed protocols were also reported.

Target Gene	Primer	Primer Sequence 5′→3′	Annealing Temperature	Reference
ITS	ITS1	TCCGTAGGTGAACCTGCGG		[32]
ITS	ITS4	TCCTCCGCTTATTGATATGC	55°	[32]
β-TUB II	Bt2a	GGTAACCAAATCGGTGCTGCTTTC		[33]
β-TUB II	Bt2b	ACCCTCAGTGTAGTGACCCTTGGC	55°	[33]
EF1α	EF1a_cibo_16F	ACCGTGCCAATACCACCAAT		This study
EF1α	EF1a_cibo_1052R	GTGCGGAGGAATTGACAAGC	59°	This study
GPDH	GPDH_cibo_316F	CGTATCGTCTTCAGAAATGCT		This study
GPDH	GPDH_cibo_1392R	CCTTGGAGATGTAGTGGAGG	57°	This study

**Table 2:** Number and size range of polymorphic bands obtained with UP-PCR amplification.

Primer	n° of Polymorphic Bands	Band Size (bp)
AA2M2	15	140–2000
AS15	25	290–3500
AS15inv	34	400–3000
AS4	23	200–2050
L15	28	250–2490
L45	19	360–2650

## Data Availability

Sequences of the 47 strains for the ITS, β-TUB II, EF1α, and GPDH regions were deposited in Genebank and accession numbers are reported in Table S4. All the research data are provided in supplementary material.

## Supplementary Materials

The following supporting information can be downloaded at https://www.mdpi.com/article/10.3390/microorganisms11112727/s1; Table S1: Sequences and annealing temperatures of primers used to assess genetic diversity within fungal populations according to van Toor et al., 2005; Table S2: Strain, origin (location with geographic coordinates, source, year of isolation), and morphotype pattern based on the description of fungal growth on four different media (CYA, MA, MEA, and PDA) for 21 days at 20 °C. * Strains tested for infection; Table S3: Morphotypes of *C. camelliae* based on the description of fungal growth on four different media (CYA, MA, MEA, and PDA) for 21 days at 20 °C not described in Saracchi et al., 2022; Table S4: Accession numbers of the nucleotide sequences for the four sequenced regions; Table S5: Matrix of UP-PCR; Phylogenetic tree calculated on the ITS (Figure S1), β-TUB II (Figure S2), EF1α (Figure S3), and GPDH (Figure S4) nucleotide sequences of the 47 *C. camelliae* strains and three different species belonging to *Sclerotiniaceae* family as outgroups, using the model K2P + R3 selected by IQ-TREE model selection program. Numbers above the nodes indicate bootstrap support values. Figure S5: UP-PCR gel images. Refs. [2,11] are cited in the supplementary materials.
