# Supplementary material for: Molecular Characterization of Ciborinia camelliae Kohn Shows Intraspecific Variability and Suggests Transcontinental Movement of the Pathogen
Source: Microorganisms. 2023 Nov 8;11(11):2727. doi: 10.3390/microorganisms11112727 (PMC10673376; doi:10.3390/microorganisms11112727)
Supplement: Supplementary file 1 [file microorganisms-11-02727-s001.zip › Supplementary Material.pdf]

Table S1. Sequences and annealing temperatures of primers used to assess genetic diversity within fungal populations according to Van Toor et al., 2005.

Primer	Primer Sequence 5′→3′	Annealing temperature (°C)
AA2M2	CTGCGACCCAGAGCGG	50
AS15	GGCTAAGCGGTCGTTA	52
AS15inv	CATTGCTGGCGAATCGG	52
AS4	TGTGGGCGCTCGACA	51
L15	GAGGGTGGCGGTTCT	52
L45	GTAAAACGACGGCCAGT	51

Table S2. Strain, origin (location with geographic coordinates, source,year of isolation), and morphotype pattern based on the description of fungal growth on four different media (CYA, MA, MEA, and PDA) for 21 days at 20°C. *Strains tested for infection.

Strain	Location	Geographic coordinates	Source	Year	Morphotype pattern	Characterization
C3	Lincoln (New Zealand)	43°39′S<br>172°29 E	Sclerotium	2017	11	This study
C5scl	Lincoln (New Zealand)	43°39′S<br>172°29 E	Sclerotium	2017	11	This study
*C5.19	Palmerston North (New Zealand)	40°21′18″ S<br>175°36′42″ E	Infected Flower	2016	11	This study
CH1	Locarno (Switzerland)	46°10′05.0000" N<br>8°47′19.0000" E	Infected Flower	2018	1	This study
CH14	Locarno (Switzerland)	46°10′05.0000" N<br>8°47′19.0000" E	Infected Flower	2018	14	This study
CH16	Locarno (Switzerland)	46°10′05.0000" N<br>8°47′19.0000" E	Infected Flower	2018	13	This study
CH7	Locarno (Switzerland)	46°10′05.0000" N<br>8°47′19.0000" E	Infected Flower	2018	1	This study
CO1	Tramezzina (CO, Italy)	45°59′10.3186" N<br>09°13′51.6598" E	Infected Flower	2019	8	[2]
CO15	Tramezzina (CO, Italy)	45°59′10.3186" N<br>09°13′51.6598" E	Infected Flower	2019	1	[2]
CO5	Tramezzina (CO, Italy)	45°59′10.3186" N<br>09°13′51.6598" E	Infected Flower	2019	1	[2]
CO6	Tramezzina (CO, Italy)	45°59′10.3186" N<br>09°13′51.6598" E	Infected Flower	2019	6	[2]
GE19	Genoa (Italy)	44°25′32.8807" N<br>08°49′09.0124" E	Sclerotium	2018	3	[2]
GE2	Genoa (Italy)	44°25′32.8807" N<br>08°49′09.0124" E	Sclerotium	2018	1	[2]
GE40	Genoa (Italy)	44°25′32.8807" N<br>08°49′09.0124" E	Sclerotium	2018	2	[2]
ITAB2	Oggebbio (VB, Italy)	45°59′47.5782" N<br>08°39′05.9659" E	Sclerotium	2016	2	[2]

*ITAC2	Oggebbio (VB, Italy)	45°59′47.5782" N 08°39′05.9659" E	Sclerotium	2016	1	[2]
ITAE1	Oggebbio (VB, Italy)	45°59′47.5782" N 08°39′05.9659" E	Sclerotium	2016	4	[2]
ITAE3	Oggebbio (VB, Italy)	45°59′47.5782" N 08°39′05.9659" E	Sclerotium	2016	1	[2]
ITAG2	Oggebbio (VB, Italy)	45°59′47.5782" N 08°39′05.9659" E	Sclerotium	2016	1	[2]
ITAH3	Oggebbio (VB, Italy)	45°59′47.5782" N 08°39′05.9659" E	Sclerotium	2016	1	[2]
ITAI2	Oggebbio (VB, Italy)	45°59′47.5782" N 08°39′05.9659" E	Sclerotium	2016	5	[2]
ITAJ1	Oggebbio (VB, Italy)	45°59′47.5782" N 08°39′05.9659" E	Sclerotium	2017	11	[2]
ITAN1	Oggebbio (VB, Italy)	45°59′47.5782" N 08°39′05.9659" E	Sclerotium	2017	1	[2]
ITAU1	Oggebbio (VB, Italy)	45°59′47.5782" N 08°39′05.9659" E	Sclerotium	2017	10	[2]
ITAV1	Oggebbio (VB, Italy)	45°59′47.5782" N 08°39′05.9659" E	Sclerotium	2017	7	[2]
LU2	Capannori (LU, Italy)	43°47′04.8685" N 10°33′47.5693" E	Sclerotium	2018	1	[2]
LU3	Capannori (LU, Italy)	43°47′04.8685" N 10°33′47.5693" E	Sclerotium	2018	9	[2]
MI2	Milan (Italy)	45°27′52.9056" N 09°13′27.0905" E	Infected Flower	2019	2	[2]
NA5	Portici (NA, Italy)	40°48′45.8813" N 14°20′10.6897" E	Infected Flower	2018	1	[2]
NT1	Nantes (France)	47°13′05.0000" N 1°33′10.0000" E	Infected Flower	2018	16	This study
NT2	Nantes (France)	47°13′05.0000" N 1°33′10.0000" E	Infected Flower	2018	7	This study
NT3	Nantes (France)	47°13′05.0000" N 1°33′10.0000" E	Infected Flower	2018	10	This study

PRC21	Dali (Yunnan, China)	25°42´01.0000" N 100°9´23.0000" E	Sclerotium	2019	10	This study
*PRC26	Dali (Yunnan, China)	25°42´01.0000" N 100°9´23.0000" E	Sclerotium	2019	10	This study
PRC40	Dali (Yunnan, China)	25°42´01.0000" N 100°9´23.0000" E	Sclerotium	2021	10	This study
PRC8	Dali (Yunnan, China)	25°42´01.0000" N 100°9´23.0000" E	Sclerotium	2019	10	This study
PT1	Porto (Portugal)	41°9.43´71.00" N 8°37´19.0300" W	Sclerotium	2017	16	This study
PT4	Porto (Portugal)	41°9.43´71.00" N 8°37´19.0300" W	Sclerotium	2017	15	This study
PT6	Porto (Portugal)	41°9.43´71.00" N 8°37´19.0300" W	Sclerotium	2017	10	This study
PT7	Porto (Portugal)	41°9.43´71.00" N 8°37´19.0300" W	Sclerotium	2017	16	This study
PT8	Porto (Portugal)	41°9.43´71.00" N 8°37´19.0300" W	Sclerotium	2017	16	This study
PT9	Porto (Portugal)	41°9.43´71.00" N 8°37´19.0300" W	Sclerotium	2017	7	This study
SPA1	Santiago di Compostela (Spain)	42°52´57.06" N 8°32´28.70" W	Sclerotium	2016	7	This study
SPA2	Santiago di Compostela (Spain)	42°52´57.06" N 8°32´28.70" W	Sclerotium	2016	7	This study
SPA3	Santiago di Compostela (Spain)	42°52´57.06" N 8°32´28.70" W	Sclerotium	2016	7	This study
SPA4	Santiago di Compostela (Spain)	42°52´57.06" N 8°32´28.70" W	Sclerotium	2016	12	This study
SPA5	Santiago di Compostela (Spain)	42°52´57.06" N 8°32´28.70" W	Sclerotium	2016	7	This study

Table S3: Morphotypes of *Ciborinia camelliae* based on the description of fungal growth on four different media (CYA, MA, MEA, and PDA) for 21 days at 20°C not described in Saracchi et al., 2022.

	**Morphotypes**				
**Strain**	CYA	**MA**	**MEA**	**PDA**	**Morphotype pattern**
CH14	B	C	F	H	14
	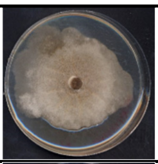	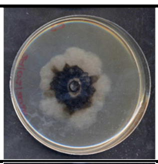	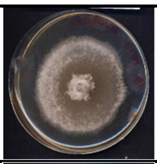	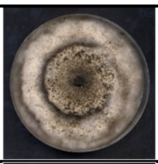	
	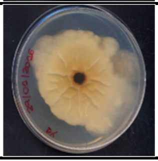	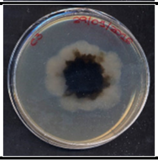	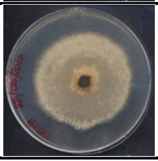	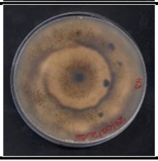	
CH16	A	C	F	L	13
	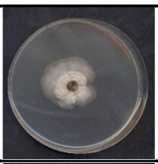	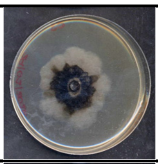	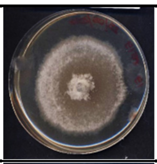	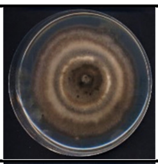	
	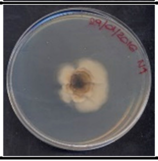	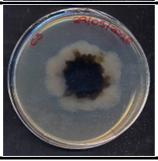	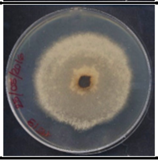	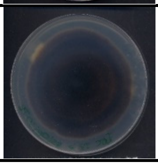	
NT1	B	C	G	H	16
	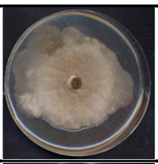	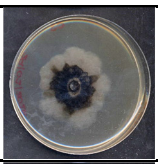	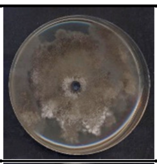	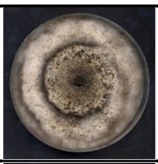	
	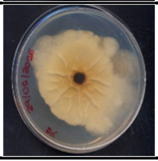	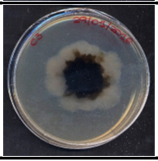	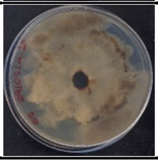	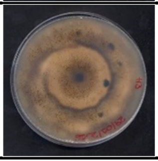	
PT1	B	C	G	H	16
	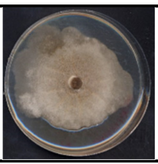	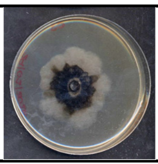	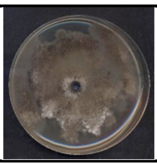	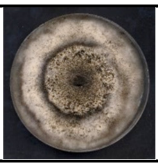	

	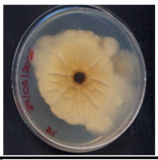	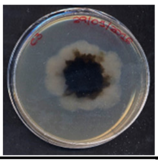	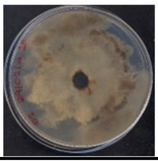	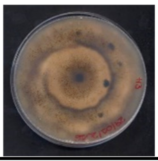	
PT4	B	C	F	I	15
	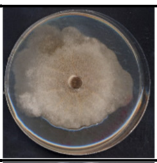	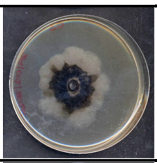	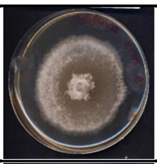	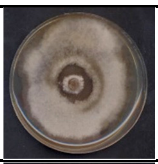	
	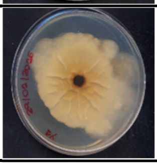	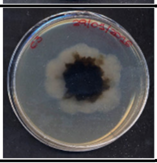	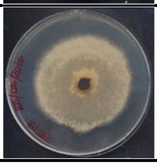	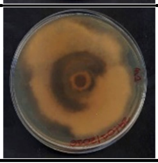	
PT7	B	C	G	H	16
	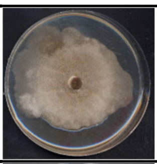	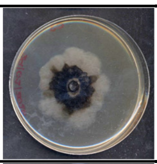	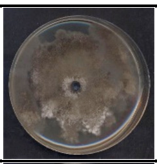	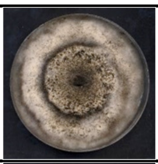	
	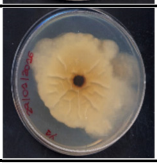	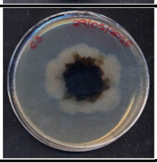	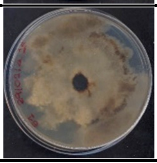	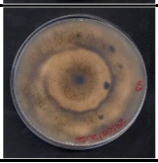	
PT8	B	C	G	H	16
	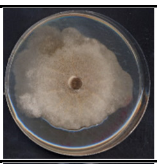	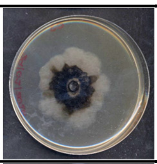	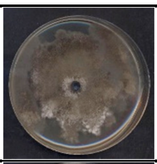	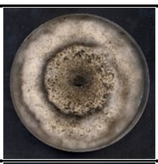	
	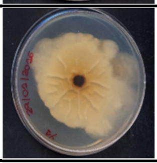	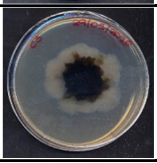	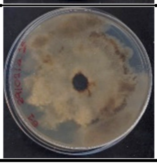	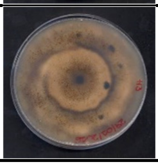	
SPA4	A	C	F	H	12
	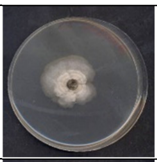	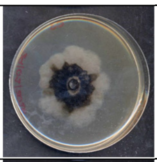	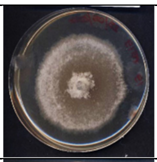	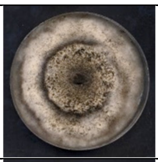	
	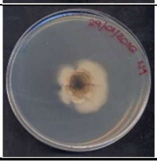	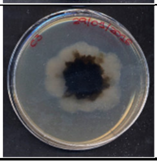	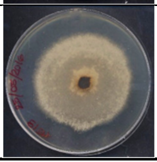	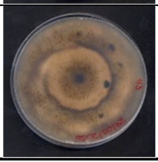	

Table S4. Accession numbers of the nucleotide sequences for the four sequenced regions: subunit II of β-tubulin (β-TUB II), elongation factor 1-α (EF1α), glycerol-3-phosphate dehydrogenase (GPDH) and nuclear ribosomal DNA internal transcribed spacer (ITS).

	Accession number			
**Strain**	**β-TUB II**	**EF1α**	**GPDH**	**ITS**
C3	MZ080164	MZ080275	MZ080276	MZ047318
C5.19	MZ080165	MZ080259	MZ080277	MZ047319
C5scl	MZ080166	MZ080274	MZ080278	MZ047320
CH1	MZ080167	MZ080227	MZ080279	MZ047321
CH7	MZ080168	MZ080258	MZ080280	MZ047322
CH14	MZ080169	MZ080226	MZ080281	MZ047323
CH16	MZ080170	MZ080257	MZ080282	MZ047324
CO1	MZ080171	MZ080256	MZ080283	MW624701
CO5	MZ080172	MZ080255	MZ080284	MW624702
CO6	MZ080173	MZ080254	MZ080285	MW624703
CO15	MZ080174	MZ080253	MZ080286	MW624704
GE2	MZ080175	MZ080252	MZ080287	MW624705
GE19	MZ080176	MZ080251	MZ080288	MW624706
GE40	MZ080177	MZ080250	MZ080289	MW624707
ITAB2	MZ080178	MZ080249	MZ080290	MW624708
ITAC2	MZ080179	MZ080248	MZ080291	MW624709
ITAE1	MZ080180	MZ080247	MZ080292	MW624710
ITAE3	MZ080181	MZ080246	MZ080293	MW624711
ITAG2	MZ080182	MZ080245	MZ080294	MW624712
ITAH3	MZ080183	MZ080220	MZ080295	MW624713
ITAI2	MZ080184	MZ080244	MZ080296	MW624714
ITAJ1	MZ080185	MZ080243	MZ080297	MW624715
ITAN1	MZ080186	MZ080225	MZ080298	MW624716
ITAU1	MZ080187	MZ080242	MZ080299	MW624717
ITAV1	MZ080188	MZ080224	MZ080300	MW624718
LU2	MZ080189	MZ080241	MZ080301	MW624719
LU3	MZ080190	MZ080223	MZ080302	MW624720
MI2	MZ080191	MZ080240	MZ080303	MW624721
NA5	MZ080192	MZ080239	MZ080304	MW624722
NT1	MZ080193	MZ080238	MZ080305	MZ047325
NT2	MZ080194	MZ080222	MZ080306	MZ047326
NT3	MZ080195	MZ080237	MZ080307	MZ047327
PRC8	MZ080198	MZ080263	MZ080327	MZ047330
PRC21	MZ080201	MZ080271	MZ080329	MZ047333
PRC26	MZ080202	MZ080270	MZ080322	MZ047334
PRC40	MZ080208	MZ080265	MZ080330	MZ047340
PT1	MZ080209	MZ080236	MZ080308	MZ047341
PT4	MZ080210	MZ080235	MZ080309	MZ047342
PT6	MZ080211	MZ080234	MZ080310	MZ047343
PT7	MZ080212	MZ080221	MZ080311	MZ047344
PT8	MZ080213	MZ080233	MZ080312	MZ047345
PT9	MZ080214	MZ080232	MZ080313	MZ047346
SPA1	MZ080215	MZ080264	MZ080314	MZ047347
SPA2	MZ080216	MZ080231	MZ080315	MZ047348
SPA3	MZ080217	MZ080230	MZ080316	MZ047349
SPA4	MZ080218	MZ080229	MZ080317	MZ047350
SPA5	MZ080219	MZ080228	MZ080318	MZ047351

Table S5. Matrix of UP-PCR

ceppi	A1	A2	A3	A4	A5	A6	A7	A8	A9	A10	A11	A12	A13	A14	A15	A16	B1	B2	B3	B4	B5	B6	B7	B8	B9	B10	B11	B12	B13	B14	B15	B16	B17	B18	B19	B20	B21	B22	B23	B24	B25	B26	B27
C3	0	0	1	0	0	0	0	0	0	0	0	0	0	0	0	0	0	0	0	0	1	0	0	0	1	0	0	1	1	0	0	1	0	0	1	1	0	0	0	0	0	0	0
C5.19	0	0	1	1	1	0	0	0	0	1	0	0	0	0	1	1	0	0	0	0	0	1	0	1	0	1	1	1	0	0	1	0	1	0	0	1	0	1	0	0	1	0	0
C5.scl	0	0	1	0	0	0	0	0	0	0	0	0	0	0	0	0	0	0	0	0	1	0	0	0	1	0	0	1	1	0	0	1	0	0	1	1	0	0	0	0	0	0	0
CH1	0	0	1	0	0	0	0	1	1	0	0	0	0	0	1	1	0	0	0	0	1	0	0	0	1	0	0	1	1	0	0	1	0	0	1	0	1	0	0	0	0	0	0
CH14	0	1	1	1	0	0	0	1	1	0	0	0	0	0	0	1	0	0	0	1	0	0	1	1	0	0	0	0	0	1	0	1	1	0	0	0	0	0	0	0	0	0	0
CH16	0	0	1	0	0	0	0	1	1	0	0	0	0	0	1	1	0	0	0	0	1	0	0	0	1	0	0	1	1	0	0	1	0	0	1	0	1	0	0	0	0	0	0
CH7	0	0	1	0	0	0	0	1	1	0	0	0	0	0	1	1	0	0	0	0	1	0	0	0	1	0	0	1	1	0	0	1	0	0	1	0	1	0	0	0	0	0	0
CO1	0	1	1	1	0	0	0	1	0	0	0	1	1	0	0	1	1	0	0	1	0	0	1	1	0	0	0	0	0	1	0	1	1	0	0	0	0	0	0	0	0	0	1
CO15	0	1	1	0	0	0	0	1	1	0	0	0	0	0	1	1	1	0	0	0	0	0	1	1	0	0	0	0	0	1	0	1	1	0	0	0	0	0	0	0	0	0	1
CO5	0	0	1	0	0	0	0	1	1	0	0	0	0	0	1	1	1	0	0	1	0	0	0	1	0	0	0	1	1	0	0	1	0	1	0	0	1	0	0	0	0	0	0
CO6	0	0	1	0	0	0	0	1	1	0	0	0	0	0	1	1	0	0	0	1	0	0	0	1	0	0	0	1	1	0	0	1	0	1	0	0	1	0	0	0	0	0	0
GE19	0	0	0	0	0	0	0	1	1	0	0	0	0	0	1	1	0	0	0	1	0	0	0	1	0	0	0	1	1	0	0	1	0	1	0	0	1	0	0	0	0	0	0
GE2	0	0	1	0	0	0	0	1	1	0	0	0	0	0	1	1	0	0	0	1	0	0	0	1	0	0	0	1	1	0	0	1	0	1	0	0	1	0	0	0	0	0	0
GE40	0	0	0	1	0	0	0	0	0	0	0	0	1	0	0	0	0	0	0	0	1	0	0	0	0	0	0	1	1	0	0	1	0	0	1	0	0	0	0	1	0	0	0
ITAB2	0	0	1	0	0	0	0	1	1	0	0	0	0	0	1	1	0	0	0	0	1	0	0	0	0	0	0	1	1	0	0	1	0	1	0	0	0	0	0	0	0	0	0
ITAC2	0	0	1	0	0	0	0	1	1	0	0	0	0	0	1	1	0	0	0	0	1	0	0	0	1	0	0	1	1	0	0	1	0	1	0	0	0	0	0	0	0	0	0
ITAE1	0	0	1	0	0	0	0	1	1	0	0	0	0	0	1	1	0	0	0	0	1	0	0	0	1	0	0	1	1	0	0	1	0	0	1	0	1	0	0	0	0	0	0
ITAE3	0	0	1	0	0	0	0	1	1	0	0	0	0	0	1	1	0	0	0	0	1	0	0	0	1	0	0	1	1	0	0	1	0	0	1	0	1	0	0	0	0	0	0
ITAG2	0	0	1	0	0	0	0	1	1	0	0	0	0	0	1	1	0	0	0	0	1	0	0	0	1	0	0	1	1	0	0	1	0	0	1	0	1	0	0	0	0	0	0
ITAH3	0	0	1	0	0	0	0	1	1	0	0	0	0	0	1	1	0	0	0	0	1	0	0	0	1	0	0	1	1	0	0	1	0	0	1	0	1	0	0	0	0	0	0
ITAI2	0	0	1	0	0	0	0	1	1	0	0	0	0	0	1	1	0	0	0	0	1	0	0	0	1	0	0	1	1	0	0	1	0	0	1	0	1	0	0	0	0	0	0
ITAJ1	0	0	1	0	0	0	0	1	1	0	0	0	0	0	1	1	0	0	0	0	1	0	0	0	1	0	0	1	1	0	0	1	0	0	1	0	1	0	0	0	0	0	0
ITAN1	0	0	1	0	0	0	0	1	1	0	0	0	0	0	1	1	0	0	0	0	1	0	0	0	1	0	0	1	1	0	0	1	0	0	1	0	1	0	0	0	0	0	0
ITAU1	0	0	1	0	0	0	0	1	1	0	0	0	0	0	1	1	0	0	0	0	1	0	0	0	1	0	0	1	1	0	0	1	0	0	1	0	1	0	0	0	0	0	0
ITAV1	0	0	1	0	0	0	0	1	1	0	0	0	0	0	1	1	0	0	0	0	1	0	0	0	1	0	0	1	1	0	0	1	0	0	1	0	0	0	0	0	0	0	0
LU2	0	0	1	0	0	0	0	1	1	0	0	0	0	0	1	1	0	0	0	0	1	0	0	0	1	0	0	1	1	0	0	1	0	0	1	0	1	0	0	0	0	0	0
LU3	0	0	1	0	0	0	0	1	1	0	0	0	0	0	1	1	0	0	0	0	1	0	0	0	1	0	0	1	1	0	0	1	0	0	1	0	1	0	0	0	0	0	0
MI2	0	0	1	0	0	0	0	1	1	0	0	0	0	0	0	1	0	0	0	0	0	0	0	0	0	1	0	1	1	0	1	1	0	0	0	0	0	0	0	0	0	0	0
NA5	0	1	1	0	0	0	0	1	1	0	0	0	0	0	1	1	0	0	0	0	0	0	0	0	0	1	0	1	1	0	1	1	0	0	0	0	0	0	0	0	0	0	0
NT1	0	1	1	0	0	0	0	0	1	1	1	1	0	0	1	0	0	1	0	0	0	0	1	0	0	0	0	0	0	1	0	1	1	0	0	0	0	0	0	0	0	0	1
NT2	0	1	1	1	0	0	0	0	1	1	1	0	0	0	1	0	1	1	0	1	0	0	0	1	0	0	0	0	0	1	0	1	1	0	0	0	0	0	0	0	0	0	1
NT3	0	0	1	1	1	0	0	0	0	1	0	0	0	0	1	1	0	0	0	0	0	1	0	1	0	1	1	1	0	0	1	0	1	0	0	1	0	1	0	0	1	0	0
PRC21	1	0	0	0	0	0	0	1	0	0	1	0	1	0	0	0	0	0	0	1	0	0	1	1	0	0	0	1	0	0	1	0	0	0	0	0	0	1	0	0	0	0	0
PRC26	0	0	0	0	0	1	1	0	0	0	1	0	0	0	0	0	0	0	0	0	0	0	1	0	1	0	0	0	0	0	0	0	1	0	0	0	0	0	0	0	0	1	0
PRC40	1	0	0	0	0	0	0	1	0	0	1	0	0	0	0	1	0	0	0	0	0	0	1	1	0	0	0	0	0	0	1	0	0	0	0	0	0	1	0	0	0	0	0
PRC8	1	0	0	0	0	0	1	0	0	0	1	0	1	0	0	0	0	0	0	1	0	0	1	1	0	0	0	1	0	0	1	0	1	0	0	0	0	1	0	0	0	0	0
PT1	0	0	1	0	0	0	0	0	1	1	0	0	0	0	0	1	1	1	0	1	0	0	0	1	0	0	0	0	0	1	0	1	1	0	0	0	0	0	0	0	0	0	1
PT4	0	0	1	0	0	0	0	0	1	1	0	0	0	0	0	0	1	1	0	1	0	0	1	1	0	0	0	0	0	1	0	1	1	0	0	0	0	0	0	0	0	0	1
PT6	0	1	1	0	0	0	0	0	1	1	0	0	0	0	0	1	1	1	0	1	0	0	1	1	0	0	0	0	0	1	0	1	1	0	0	0	0	0	0	0	0	0	1
PT7	0	0	1	0	0	0	0	1	1	0	0	0	0	0	0	0	0	0	0	0	1	0	0	0	1	0	0	1	1	0	0	1	0	0	1	0	1	0	0	0	0	0	0
PT8	0	1	1	1	0	0	0	0	1	1	0	1	0	0	0	0	1	1	0	1	0	0	1	1	0	0	0	0	0	1	0	1	1	0	0	0	0	0	0	0	0	0	1
PT9	0	0	1	0	0	0	0	1	1	0	0	0	0	0	1	1	0	0	0	0	1	0	0	0	1	0	0	1	1	0	0	1	0	0	1	0	1	0	0	0	0	0	0
SPA1	0	1	1	0	0	0	0	0	1	1	1	1	0	0	0	0	0	1	0	1	0	0	1	1	0	0	0	0	0	1	0	1	1	0	0	0	0	0	0	0	0	0	1
SPA2	0	1	1	1	0	0	0	0	1	1	1	0	0	0	0	0	0	1	0	1	0	0	1	1	0	0	0	0	0	1	0	1	1	0	0	0	0	0	0	0	0	0	1
SPA3	0	0	1	0	0	0	0	0	1	1	0	0	0	0	0	1	0	0	0	1	0	0	1	1	0	0	0	0	0	1	0	1	1	0	0	0	0	0	0	0	0	0	0
SPA4	0	0	1	0	0	0	0	0	1	1	0	0	0	0	0	1	0	1	0	1	0	0	1	1	0	0	0	0	1	1	0	1	1	0	0	0	0	0	0	0	0	0	1
SPA5	0	0	1	0	0	0	0	0	1	1	0	0	0	0	0	1	0	1	0	1	0	0	1	1	0	1	0	0	1	1	0	1	1	0	0	0	0	0	0	0	0	0	0
	3	10	41	8	2	1	2	29	37	13	8	4	4	0	27	34	8	10	0	18	21	2	16	22	20	5	2	31	29	14	7	41	18	6	19	4	19	5	0	1	2	1	11

	ceppi	C1	C2	C3	C4	C5	C6	C7	C8	C9	C10	C11	C12	C13	C14	C15	C16	C17	C18	C19	C20	C21	C22	C23	C24	C25	C26	C27	C28	C29	C30	C31	C32	C33	C34
1	C3	0	1	0	0	0	0	0	0	0	0	1	0	0	0	0	1	0	0	0	0	0	1	1	0	0	1	1	0	0	1	0	0	0	0
2	C5.scl	0	1	0	0	0	1	0	0	0	0	1	0	0	0	0	1	0	0	0	0	0	1	1	0	0	1	1	0	0	1	0	0	0	0
3	CH1	0	1	0	0	0	0	1	0	1	0	0	0	1	0	1	0	0	0	0	0	0	1	1	0	0	1	1	0	0	1	0	0	0	0
4	CH7	0	1	0	0	0	0	1	0	1	0	0	0	1	0	1	0	0	0	0	0	0	1	1	0	0	1	1	0	0	1	0	0	0	0
5	CH16	0	1	0	0	0	0	1	0	1	0	0	0	1	0	1	0	0	0	0	0	0	1	1	0	0	1	1	0	0	1	0	0	0	0
6	PT7	0	1	0	0	0	1	0	0	1	0	0	0	1	0	1	0	0	0	0	0	0	1	1	0	0	1	1	0	0	1	0	0	0	0
7	PT9	0	1	0	0	0	1	0	0	1	0	0	0	1	0	1	0	0	0	0	0	0	1	1	0	0	1	1	0	0	1	0	0	0	0
8	CO5	0	1	0	0	0	0	1	0	1	0	0	0	1	0	1	0	0	0	0	0	0	1	1	0	0	1	1	0	0	1	0	0	0	0
9	CO6	1	1	0	0	0	0	1	0	1	0	0	0	1	0	1	0	0	0	0	0	0	1	1	0	0	1	1	0	0	1	0	0	0	0
10	GE2	1	1	0	0	0	0	1	0	1	0	0	0	1	0	1	0	0	0	0	0	0	1	1	0	0	1	1	0	0	1	0	0	0	0
11	GE19	0	1	0	0	0	0	1	0	1	0	0	0	1	0	1	0	0	0	0	0	0	1	1	0	0	1	1	0	0	1	0	0	0	0
12	ITAB2	1	1	0	0	0	1	1	0	1	0	0	0	1	0	1	0	0	0	0	0	0	1	1	0	0	1	1	0	0	1	0	0	0	0
13	ITAE1	1	1	0	0	0	0	1	0	1	0	0	0	1	0	1	0	0	0	0	0	0	1	1	0	0	1	1	0	0	1	0	0	0	0
14	ITAE3	1	1	0	0	0	0	1	0	1	0	0	0	1	0	1	0	0	0	0	0	0	1	1	0	0	1	1	0	0	1	0	0	0	0
15	ITAG2	1	1	0	0	0	0	1	0	1	0	0	0	1	0	1	0	0	0	0	0	0	1	1	0	0	1	1	0	0	1	0	0	0	0
16	ITAI2	1	1	0	0	0	0	1	0	1	0	0	0	1	0	1	0	0	0	0	0	0	1	1	0	0	1	1	0	0	1	0	0	0	0
17	LU2	0	1	0	0	0	0	1	0	1	0	0	0	1	0	1	0	0	0	0	0	0	1	1	0	0	1	1	0	0	1	0	0	0	0
18	LU3	0	1	0	0	0	0	1	0	1	0	0	0	1	0	1	0	0	0	0	0	0	1	1	0	0	1	1	0	0	1	0	0	0	0
19	ITAJ1	1	1	0	0	0	0	1	0	1	0	0	0	1	0	1	0	0	0	0	0	0	1	1	0	0	1	1	0	0	1	0	0	0	0
20	ITAN1	1	1	0	0	0	0	1	0	1	0	0	0	1	0	1	0	0	0	0	0	0	1	1	0	0	1	1	0	0	1	0	0	0	0
21	ITAH3	0	1	0	0	0	0	1	0	1	0	0	0	1	0	0	0	0	0	0	0	0	1	1	0	0	1	1	0	0	1	0	0	0	0
22	ITAU1	1	1	0	0	0	0	1	0	1	0	0	0	1	0	1	0	0	0	0	0	0	1	1	0	0	1	1	0	0	1	0	0	0	0
23	ITAV1	0	1	0	0	0	1	0	0	1	0	0	0	1	0	1	0	0	0	0	0	0	1	1	0	0	1	1	0	0	1	0	0	0	0
24	ITAC2	1	1	0	0	0	0	1	0	1	0	0	0	1	0	0	0	0	0	0	0	0	1	1	0	0	1	1	0	0	1	0	0	0	0
25	CH14	1	1	0	0	0	0	1	0	0	1	0	0	0	0	0	0	0	1	1	0	0	1	0	0	0	0	0	0	0	0	0	0	0	0
26	CO1	1	1	0	0	0	0	1	0	0	1	0	0	0	0	0	0	0	0	1	1	0	1	0	0	0	0	0	0	0	0	0	0	0	0
27	CO15	0	1	0	0	0	0	1	0	1	0	0	1	0	0	0	0	0	0	1	0	0	1	0	0	0	0	0	0	0	0	0	0	0	0
28	MI2	0	1	0	0	0	0	1	0	0	1	0	1	0	0	0	0	0	1	0	0	0	0	0	0	0	0	0	0	0	0	0	0	0	0
29	NA5	0	0	1	0	0	0	1	0	0	1	0	0	0	0	0	0	0	1	0	0	0	0	0	0	0	0	0	0	0	0	0	0	0	1
30	NT1	0	1	0	0	0	0	1	0	1	0	0	0	1	0	0	0	0	0	0	0	0	0	1	0	1	1	0	0	0	0	0	0	1	0
31	SPA4	0	1	0	0	0	0	1	0	1	0	0	0	1	0	0	0	0	0	0	0	0	0	1	0	1	1	0	0	0	0	0	0	1	1
32	SPA5	0	1	0	0	0	0	1	0	1	0	0	0	0	0	0	0	0	0	0	0	0	0	1	0	1	1	1	0	0	0	0	0	1	1
33	NT2	0	1	0	0	0	0	1	0	1	0	0	0	1	0	0	0	0	0	0	0	0	0	1	0	1	0	0	0	0	0	0	0	0	0
34	SPA1	0	1	0	0	0	0	1	0	1	0	0	0	1	0	0	0	0	0	0	0	0	0	1	0	1	0	0	0	0	0	0	0	0	1
35	SPA2	0	1	0	0	0	0	1	0	1	0	0	0	1	0	0	0	0	0	0	0	0	0	1	0	1	0	0	0	0	0	0	0	0	0
36	PT1	0	1	0	0	0	0	1	0	1	0	0	0	1	0	0	0	0	0	0	0	0	0	1	0	1	0	0	0	0	0	0	0	0	0
37	PT4	0	1	0	0	0	0	1	0	1	0	0	0	1	0	0	0	0	0	0	0	0	0	1	0	1	0	0	0	0	0	0	0	0	0
38	PT8	0	1	0	0	0	0	1	0	1	0	0	0	1	0	0	0	0	0	0	0	0	0	1	0	1	0	0	0	0	0	0	0	0	0
39	PT6	0	1	0	0	0	0	1	0	1	0	0	0	1	0	0	0	0	0	0	0	0	0	1	0	1	0	0	0	0	0	0	0	0	0
40	SPA3	0	1	0	0	0	0	1	0	1	0	0	0	1	0	0	0	0	0	0	0	0	0	1	0	1	0	0	0	0	0	0	0	0	0
41	GE40	0	0	0	1	0	0	0	1	0	0	0	0	0	0	0	0	0	0	0	1	0	0	0	0	0	1	1	0	0	1	0	0	0	0
42	PRC21	0	1	0	0	1	0	1	0	1	0	1	0	0	0	1	0	0	0	0	0	0	0	1	0	1	0	0	1	0	0	1	0	0	0
43	PRC40	0	1	0	0	1	0	1	0	1	0	1	0	0	0	0	0	0	0	0	0	1	0	1	0	1	0	0	1	0	0	0	0	0	0
44	PRC8	1	1	0	0	1	0	1	0	1	0	1	0	0	0	1	0	0	0	0	0	1	0	0	0	1	1	1	1	0	1	0	1	0	0
45	PRC26	1	1	0	1	1	0	0	0	0	1	0	1	0	0	0	0	0	0	0	0	1	0	1	1	1	1	0	0	1	0	0	0	0	0
46	C5.19	0	1	1	0	0	0	0	1	0	0	1	0	0	1	0	0	1	0	0	0	1	0	1	0	0	0	0	0	0	0	1	1	1	0
47	NT3	0	1	1	0	0	0	0	1	0	0	1	0	0	1	0	0	1	0	0	0	1	0	1	0	0	0	0	0	0	0	1	1	1	0
		15	45	3	2	4	5	38	3	37	5	7	3	32	2	22	2	2	3	3	2	5	27	40	1	15	30	27	3	1	26	3	3	5	4

ceppi	D1	D2	D3	D4	D5	D6	D7	D8	D9	D10	D11	D12	D13	D14	D15	D16	D17	D18	D19	D20	D21	D22	D23	E1	E2	E3	E4	E5	E6	E7	E8	E9
C3	0	0	0	0	1	0	0	1	0	1	0	0	0	0	0	1	0	1	0	0	1	0	1	0	0	0	0	0	0	0	0	0
C5.19	0	0	1	0	0	0	1	0	0	0	0	0	1	0	0	0	0	0	0	0	1	1	0	0	0	0	1	0	0	1	0	0
C5.scl	1	0	1	0	1	0	1	0	1	0	1	0	0	0	0	1	1	0	0	0	1	1	1	0	0	0	1	0	0	0	0	1
CH1	1	1	1	0	1	0	1	0	1	0	0	0	1	0	0	1	0	0	1	0	1	1	0	1	0	0	1	0	0	0	0	0
CH14	1	0	1	0	1	0	1	0	1	0	0	0	1	0	1	0	1	0	0	1	0	1	0	0	0	0	1	0	0	1	0	1
CH16	1	1	1	0	1	0	1	0	1	0	0	0	1	0	0	1	0	1	0	0	1	1	0	1	0	0	1	0	0	0	0	0
CH7	1	1	1	0	1	0	1	0	1	0	0	0	1	0	0	1	0	0	0	0	1	1	1	1	0	0	1	1	0	0	0	0
CO1	1	0	1	0	1	0	1	0	1	0	0	0	1	0	1	0	1	0	0	1	0	1	0	0	0	0	1	0	0	1	0	1
CO15	0	0	1	0	1	0	1	0	1	0	0	0	1	0	1	0	1	0	0	1	0	1	0	0	0	0	1	0	1	1	0	1
CO5	1	1	1	0	1	0	1	0	1	0	0	0	0	0	1	0	1	0	0	1	0	1	0	1	0	0	1	0	1	1	0	0
CO6	1	1	1	0	0	1	0	1	0	1	0	1	0	0	1	0	1	0	0	1	0	1	1	1	0	0	1	0	1	1	0	1
GE19	1	1	1	0	1	0	0	1	0	1	0	1	0	0	1	0	0	0	0	0	1	0	0	1	0	0	1	1	1	1	0	1
GE2	1	1	1	0	1	0	0	1	0	1	0	1	0	0	1	0	0	0	0	1	0	1	0	1	0	0	1	1	0	1	0	0
GE40	0	0	0	0	1	0	0	0	0	0	0	1	0	1	0	0	0	0	0	1	0	0	0	1	0	0	1	1	0	1	0	1
ITAB2	1	1	1	0	1	0	1	0	1	0	0	1	0	0	1	0	1	0	0	1	0	1	0	1	0	0	1	0	1	1	0	1
ITAC2	1	1	1	0	1	0	1	0	1	0	0	1	0	0	1	0	0	1	0	1	0	1	0	1	0	1	1	0	0	0	1	0
ITAE1	1	1	1	0	1	0	1	0	1	0	0	1	0	0	1	0	1	0	0	1	0	1	0	1	0	0	1	0	0	0	0	1
ITAE3	1	1	1	0	1	0	1	0	1	0	0	1	0	0	1	0	1	0	0	1	0	1	0	1	0	0	1	1	0	1	0	0
ITAG2	1	1	1	0	1	0	1	0	1	0	0	1	0	0	1	0	1	0	0	1	0	1	0	1	0	0	1	0	0	1	1	0
ITAH3	1	1	1	0	1	0	1	0	1	0	0	1	0	0	1	0	0	1	0	1	0	1	0	1	0	0	1	0	1	1	0	1
ITAI2	1	1	1	0	1	0	1	0	1	0	0	1	0	0	1	0	1	0	0	1	0	1	0	1	0	0	1	0	0	1	0	0
ITAJ1	1	1	1	0	1	0	1	0	1	0	0	1	0	0	1	0	1	0	0	1	0	1	0	1	0	0	1	0	1	1	0	1
ITAN1	0	0	0	0	1	0	1	0	1	0	0	1	0	0	1	0	0	1	0	1	0	1	0	1	0	0	1	0	1	1	0	1
ITAU1	0	0	0	0	1	0	1	0	1	0	0	1	0	0	1	0	1	0	0	1	0	1	1	1	0	0	1	0	0	1	0	0
ITAV1	1	1	1	0	1	0	1	0	0	0	0	1	0	0	1	0	1	0	0	1	0	1	0	1	0	0	1	1	0	1	0	0
LU2	1	1	1	0	0	0	1	0	1	0	0	1	0	0	1	0	1	0	0	1	0	1	0	1	0	0	1	0	0	1	0	0
LU3	1	1	1	0	1	0	1	0	1	0	0	1	0	0	1	0	1	0	0	1	0	1	0	1	0	0	1	0	1	1	0	0
MI2	0	0	1	0	1	0	1	0	1	0	0	0	1	0	1	0	1	0	0	1	0	1	0	0	0	0	1	0	1	0	0	1
NA5	0	0	1	0	1	0	1	0	1	0	0	0	1	0	1	0	1	0	0	1	0	1	0	0	0	0	1	0	1	0	0	1
NT1	1	0	0	0	1	0	1	0	0	0	0	0	1	0	1	0	1	0	0	1	0	1	1	0	0	0	1	0	1	0	0	1
NT2	1	1	0	0	1	0	1	0	1	0	0	0	1	0	1	0	1	0	0	1	0	1	1	0	0	0	1	0	0	1	0	1
NT3	0	0	1	0	0	0	1	0	0	0	0	0	1	0	0	0	0	0	0	0	1	1	0	0	0	0	1	0	0	1	0	0
PRC21	0	1	0	0	1	0	1	0	1	0	0	0	0	0	0	0	1	0	0	1	0	1	0	0	0	0	1	0	1	1	0	0
PRC26	0	1	1	1	0	0	1	0	1	0	0	0	0	0	0	0	0	0	0	0	0	0	0	0	1	0	1	0	1	1	0	0
PRC40	0	1	0	0	1	0	1	0	1	0	0	0	0	0	0	0	1	0	0	1	0	1	0	0	0	0	1	0	1	1	0	0
PRC8	0	1	0	0	1	0	1	0	1	0	0	0	0	0	1	0	1	0	0	1	0	1	0	0	0	0	0	0	1	1	0	0
PT1	0	0	1	0	1	0	1	0	1	0	0	0	1	0	1	0	1	0	0	1	0	1	0	0	0	0	1	0	0	1	0	1
PT4	1	0	1	0	1	0	0	0	1	0	0	0	1	0	1	0	1	0	0	1	0	1	1	0	0	0	1	0	1	1	0	1
PT6	1	0	1	0	1	0	0	0	1	0	0	0	1	0	1	0	0	0	0	1	0	1	1	0	0	0	1	0	0	1	0	1
PT7	0	0	1	0	1	0	0	1	0	0	0	0	1	0	0	1	0	0	1	0	1	1	1	1	0	0	1	0	0	0	0	0
PT8	1	0	1	0	1	0	0	0	1	0	0	0	1	0	1	0	1	0	0	1	0	1	1	0	0	0	1	0	1	1	0	1
PT9	0	0	1	0	1	0	0	1	0	0	0	0	0	0	0	1	0	0	1	0	1	1	0	1	0	0	1	1	0	1	0	0
SPA1	0	0	0	0	1	0	1	0	0	0	0	0	1	0	1	0	0	0	0	1	0	1	1	0	0	0	1	0	0	1	0	1
SPA2	0	0	0	0	1	0	1	0	1	0	0	0	1	0	1	0	0	0	0	1	0	1	1	0	0	0	1	0	0	1	0	1
SPA3	0	0	1	0	1	0	1	0	1	0	0	0	1	0	1	0	0	0	0	1	0	1	1	0	0	0	1	0	0	1	0	1
SPA4	0	0	0	0	1	0	1	0	0	0	0	0	1	0	1	0	0	0	0	1	0	1	1	0	0	0	1	0	1	0	0	1
SPA5	0	0	0	0	1	0	1	0	0	0	0	0	1	0	1	0	0	0	0	1	0	1	1	1	0	0	1	0	1	0	0	0
	26	23	34	1	42	1	37	6	33	4	1	17	22	1	34	7	26	5	3	36	10	43	16	24	1	1	45	7	20	34	2	24

	ceppi	E10	E11	E12	E13	E14	E15	E16	E17	E18	E19	E20	E21	E22	E23	E24	E25	E26	E27	E28	F1	F2	F3	F4	F5	F6	F7	F8	F9	F10	F11	F12	F13	F14	F15	F16	F17	F18	F19
1	C3	0	0	0	0	1	1	0	0	0	0	0	1	0	0	0	0	0	0	0	0	0	0	0	0	0	0	1	0	0	1	1	0	0	0	0	0	1	0
2	C5.scl	1	0	0	1	1	0	0	0	1	0	0	0	0	0	0	0	0	0	0	0	0	0	0	0	1	0	1	0	0	0	1	0	0	0	0	0	1	0
3	CH1	0	0	1	0	1	0	1	0	0	0	0	0	1	0	0	0	0	0	0	0	0	0	1	0	0	0	1	0	0	1	1	0	0	0	0	1	0	1
4	CH7	0	0	0	0	1	0	1	0	0	0	0	0	1	0	0	0	0	0	0	0	0	0	1	0	0	0	1	0	0	1	1	0	0	0	0	1	0	0
5	CH16	0	0	0	0	1	0	1	0	0	0	0	0	0	0	0	0	0	0	0	0	0	0	1	0	0	0	1	0	0	1	1	0	0	0	0	0	0	0
6	PT7	0	0	1	0	1	0	1	0	0	0	0	0	1	0	0	0	0	0	0	0	1	0	1	0	0	0	1	0	0	1	1	0	0	0	0	0	0	1
7	PT9	0	0	1	0	1	0	0	0	0	0	0	0	1	0	0	0	0	0	0	0	1	0	1	0	0	0	1	0	0	1	1	0	0	0	0	0	0	0
8	CO5	0	0	0	0	0	1	0	0	0	0	0	0	0	0	0	0	0	0	0	0	0	0	1	0	0	1	0	0	1	0	0	0	0	0	0	0	0	0
9	CO6	1	0	0	1	0	1	0	0	1	0	0	1	0	1	0	0	0	0	0	0	0	0	1	0	0	1	0	0	1	0	0	0	0	0	0	0	1	0
10	GE2	0	0	0	1	0	1	0	0	0	0	0	0	0	1	0	0	0	0	0	0	1	0	1	0	0	1	0	1	1	0	0	0	0	0	0	0	0	1
11	GE19	1	0	0	0	0	0	0	1	0	1	0	0	0	0	0	0	0	0	0	0	0	0	1	0	0	1	0	0	0	0	0	0	0	0	0	0	0	0
12	ITAB2	1	0	1	0	1	0	0	0	0	0	0	0	1	0	0	0	0	0	0	0	1	1	1	0	0	1	0	1	1	0	0	0	0	0	0	0	1	1
13	ITAE1	0	0	0	0	1	0	0	0	0	0	0	0	1	0	0	0	0	0	0	0	0	0	1	0	0	1	0	1	1	0	0	0	0	0	0	0	0	0
14	ITAE3	0	0	0	0	1	0	0	0	0	0	0	0	1	0	0	0	0	0	0	0	0	0	1	0	0	1	0	1	1	0	0	0	0	0	1	0	0	0
15	ITAG2	0	0	1	0	1	0	0	0	0	0	0	0	0	0	0	0	0	0	0	0	1	0	1	0	0	1	0	0	1	0	0	0	0	0	0	0	0	0
16	ITAI2	1	0	0	0	1	0	0	0	0	0	0	1	0	0	0	0	0	0	0	0	1	0	1	0	0	1	0	1	1	0	0	0	0	0	0	0	0	0
17	LU2	0	1	1	0	1	0	0	0	0	0	0	1	0	0	0	0	0	0	0	0	0	0	1	0	0	1	0	1	1	0	0	0	0	0	1	0	0	0
18	LU3	0	0	1	0	1	0	0	0	0	0	0	1	0	0	0	0	0	0	0	0	0	0	1	0	0	1	0	1	1	0	0	0	0	0	1	0	0	1
19	ITAJ1	1	0	0	1	1	0	1	0	1	0	0	1	0	0	0	0	0	0	0	0	1	0	1	0	0	1	0	1	1	0	0	0	0	0	1	0	0	1
20	ITAN1	1	0	0	1	1	0	1	0	1	0	0	1	1	0	0	0	0	0	0	0	0	0	1	0	0	1	0	1	1	0	0	0	0	0	1	0	0	1
21	ITAH3	1	1	1	0	1	1	0	0	1	0	0	0	0	0	0	0	0	0	0	0	0	0	1	0	0	1	0	1	1	0	0	0	0	0	0	0	0	0
22	ITAU1	1	1	0	0	1	0	1	0	0	0	0	1	0	0	0	0	0	0	0	0	0	1	1	0	0	1	0	1	1	0	0	0	0	0	0	0	1	0
23	ITAV1	1	1	0	0	1	0	0	0	0	0	0	1	0	0	0	0	0	0	0	0	0	1	1	0	0	1	0	0	1	0	0	0	0	0	0	0	1	1
24	ITAC2	0	0	0	0	1	0	0	0	0	0	0	0	1	0	0	0	0	0	0	0	0	0	1	0	0	1	0	1	1	0	0	0	0	0	1	0	0	0
25	CH14	1	0	1	0	0	1	0	0	1	0	0	1	0	0	0	1	0	0	0	0	0	0	1	0	1	1	0	0	0	0	1	0	0	0	0	0	0	1
26	CO1	1	0	1	0	0	1	0	0	1	0	0	1	0	0	0	1	0	0	0	0	0	0	1	0	1	1	0	1	0	0	1	1	0	0	1	0	0	0
27	CO15	1	0	1	0	0	1	0	0	1	0	0	1	0	0	0	1	0	0	0	0	1	0	1	0	0	1	0	0	0	0	1	1	0	0	1	0	0	1
28	MI2	1	0	1	1	0	1	0	0	1	0	0	1	0	0	0	0	0	0	0	0	0	0	1	0	0	1	0	0	1	0	1	1	0	0	0	0	0	0
29	NA5	1	1	1	1	0	1	0	0	1	0	0	1	0	0	1	0	0	0	0	1	0	0	1	0	0	1	0	0	0	0	1	1	0	0	1	0	0	1
30	NT1	1	0	1	1	0	1	0	0	0	1	0	0	1	0	0	0	0	0	0	0	1	1	1	0	0	1	0	0	1	0	0	1	1	0	1	0	0	0
31	SPA4	1	0	1	1	0	1	0	0	0	1	0	0	1	0	0	1	0	0	0	0	0	1	1	0	0	1	0	0	1	0	0	1	1	0	1	0	0	0
32	SPA5	1	0	1	1	0	1	0	0	0	1	0	0	1	0	0	1	0	0	0	0	0	1	1	0	0	1	0	1	1	0	0	1	1	0	0	0	0	0
33	NT2	1	0	1	0	0	1	0	0	0	1	0	0	1	0	1	0	1	0	0	0	1	0	1	1	0	1	0	0	0	0	0	1	1	0	1	0	0	0
34	SPA1	1	0	1	0	0	1	0	0	0	1	0	0	1	0	0	0	1	0	0	0	0	0	1	1	0	1	0	0	1	0	0	1	1	0	1	0	1	0
35	SPA2	1	0	1	0	0	1	0	0	0	1	0	0	1	0	0	0	1	0	0	0	0	0	1	1	0	1	0	0	1	0	0	1	1	0	1	0	1	0
36	PT1	1	0	1	0	0	0	0	0	0	0	0	0	0	0	0	0	0	0	0	0	0	0	1	1	0	1	0	0	1	0	0	1	1	0	0	0	0	0
37	PT4	1	0	1	0	0	1	0	0	0	1	0	0	0	0	0	0	0	0	0	0	0	0	1	1	0	1	0	0	1	0	0	1	1	0	1	0	0	0
38	PT8	1	0	1	0	0	1	0	0	0	1	0	0	0	0	0	0	0	0	0	0	0	0	1	1	0	1	0	0	1	0	0	1	1	0	1	0	0	0
39	PT6	1	0	1	0	0	1	0	0	0	1	0	0	0	0	0	0	0	0	0	0	0	0	1	1	0	1	0	0	1	0	0	1	1	0	0	1	1	0
40	SPA3	1	0	1	1	0	1	0	0	0	1	0	0	1	0	0	0	0	0	0	0	0	1	1	1	0	1	0	0	1	0	0	1	1	0	1	0	0	0
41	GE40	0	0	1	0	1	0	0	0	0	0	0	0	0	0	0	1	0	1	1	0	1	0	1	0	1	0	0	1	1	0	0	0	0	0	0	0	0	0
42	PRC21	0	1	0	0	0	1	0	0	0	1	0	0	1	0	0	0	0	0	0	1	1	0	0	0	1	0	0	1	0	0	0	0	1	1	0	0	0	1
43	PRC40	0	1	0	0	0	0	0	0	0	1	1	0	1	0	0	0	0	0	0	1	1	0	0	0	1	0	0	1	1	0	0	0	1	1	0	0	0	1
44	PRC8	0	1	0	0	0	1	0	0	0	1	1	0	1	0	0	0	0	0	0	1	1	0	0	0	1	0	0	1	1	0	0	0	0	1	0	0	0	1
45	PRC26	0	0	0	0	0	0	0	0	0	0	0	0	0	0	0	0	0	0	0	0	1	1	0	0	1	0	1	0	0	1	0	0	0	0	0	1	0	0
46	C5.19	0	0	0	0	1	0	1	0	0	0	0	0	1	0	0	1	0	1	0	1	0	0	0	0	0	0	0	1	0	0	0	1	1	0	0	0	0	0
47	NT3	0	0	0	0	1	0	1	0	0	0	0	0	1	0	0	1	0	1	0	1	0	0	0	0	0	0	0	1	0	0	0	1	1	0	0	0	0	0
		26	8	25	11	23	22	9	1	10	14	2	14	21	2	2	8	3	3	1	6	15	8	39	8	8	33	8	20	30	7	12	17	15	3	17	4	9	14

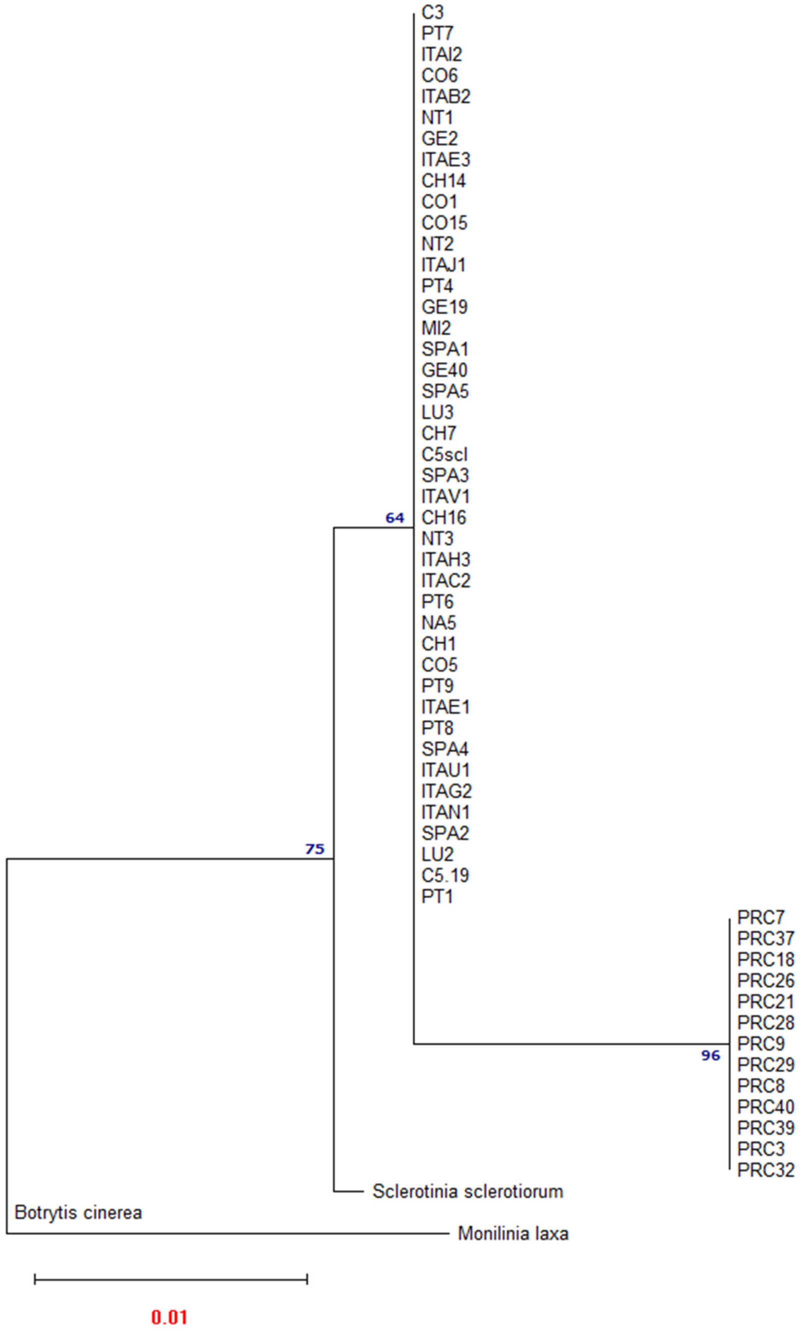

Figure S1. Phylogenetic tree calculated on the ITS nucleotide sequences of the 47 *C. camelliae* strains and three different species belonging to *Sclerotiniaceae* family as outgroups, using the model K2P+R3 selected by IQ-TREE model selection program. Numbers above the nodes indicate bootstrap support values.

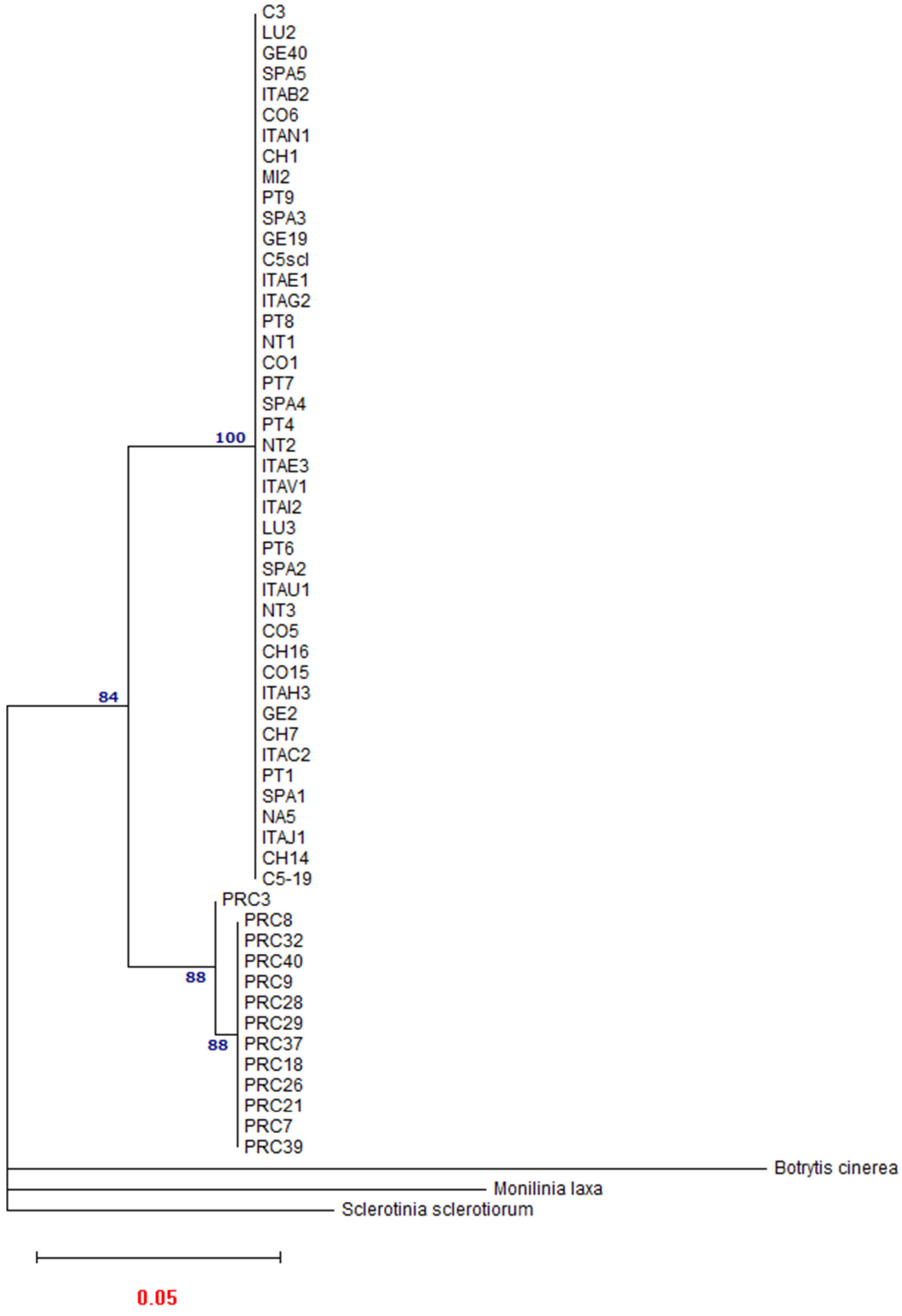

Figure S2. Phylogenetic tree calculated on the β-TUB II nucleotide sequences of the 47 *C. camelliae* strains and three different species belonging to *Sclerotiniaceae* family as outgroups, using the model K2P+R3 selected by IQ-TREE model selection program. Numbers above the nodes indicate bootstrap support values.

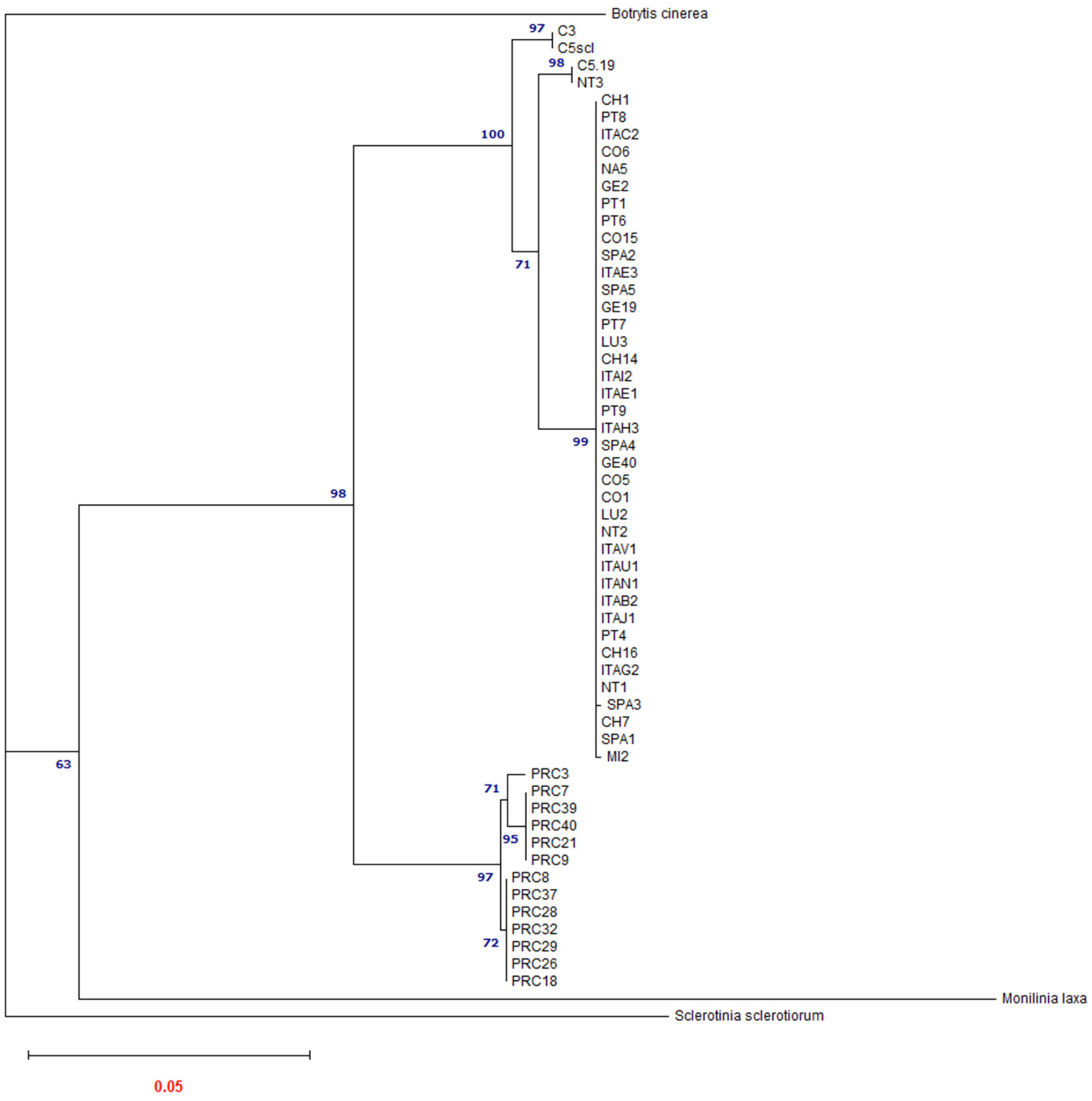

Figure S3. Phylogenetic tree calculated on the EF1α, nucleotide sequences of the 47 *C. camelliae* strains and three different species belonging to *Sclerotiniaceae* family as outgroups, using the model K2P+R3 selected by IQ-TREE model selection program. Numbers above the nodes indicate bootstrap support values.

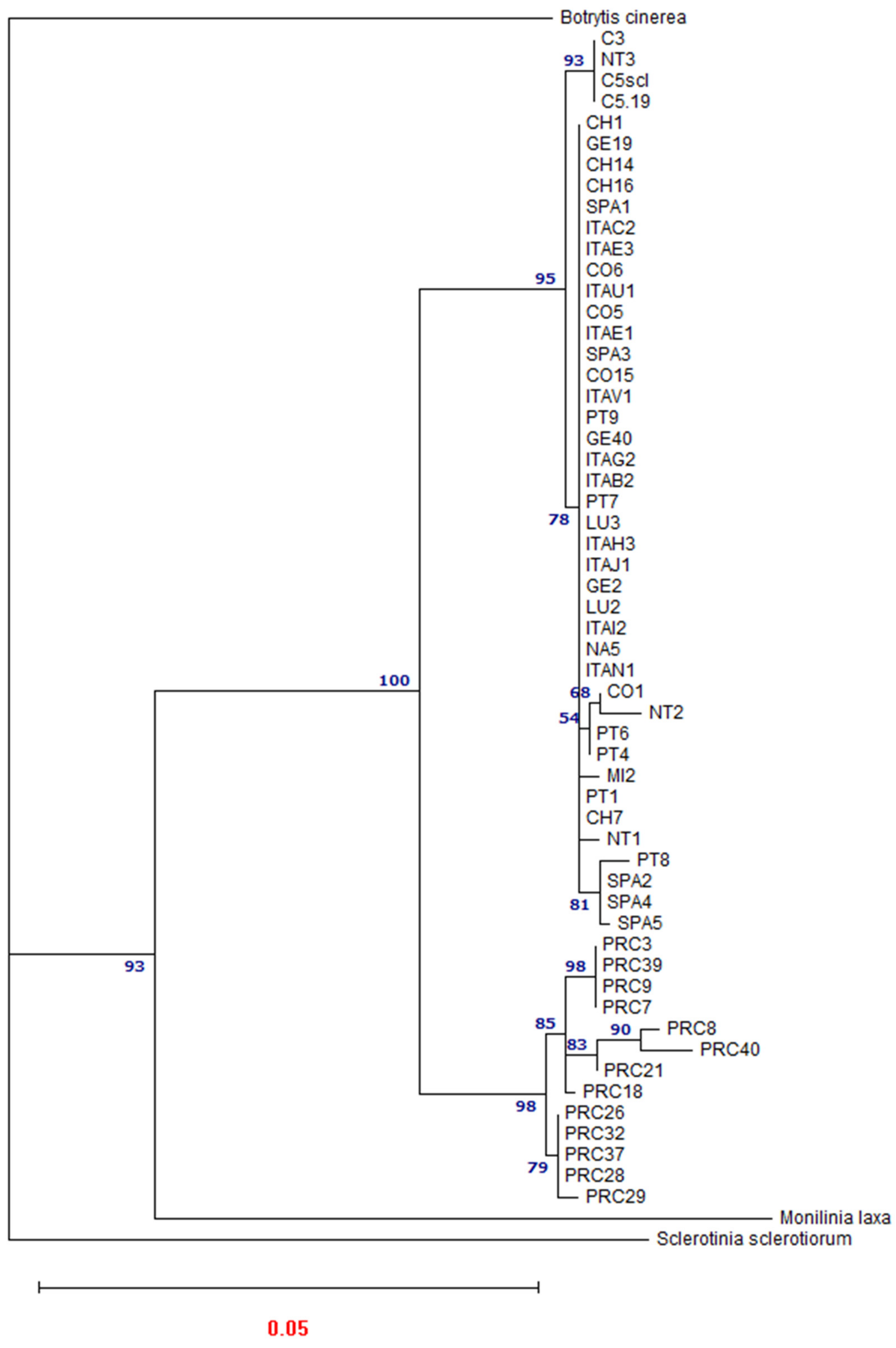

Figure S4. Phylogenetic tree calculated on GPDH nucleotide sequences of the 47 *C. camelliae* strains and three different species belonging to *Sclerotiniaceae* family as outgroups, using the model K2P+R3 selected by IQ-TREE model selection program. Numbers above the nodes indicate bootstrap support values.

References

van Toor, R.F.; Ridgway, H.J.; Butler, R.C.; VJaspers, M.; Stewart, A. Assessment of Genetic Diversity in Isolates of *Ciborinia Camelliae* Kohn from New Zealand and the United States of America. *Australasian Plant Pathology* **2005**, *34*, 319–325, doi:10.1071/AP05040.

Saracchi, M., Colombo, E. M., Locati, D., Valenti, I., Corneo, A., Cortesi, P., Kunova, A., & Pasquali, M. (2022). Morphotypes of Ciborinia camelliae Kohn infecting camellias in Italy. *Journal of Plant Pathology*, *104*(2), 761–768. https://doi.org/10.1007/s42161-022-01040-2
