# Supplementary material for: Molecular Characterization of Ciborinia camelliae Kohn Shows Intraspecific Variability and Suggests Transcontinental Movement of the Pathogen
Source: Microorganisms. 2023 Nov 8;11(11):2727. doi: 10.3390/microorganisms11112727 (PMC10673376; doi:10.3390/microorganisms11112727)
Supplement: Supplementary file 1 [file microorganisms-11-02727-s001.zip › up-pcr gel images.pdf]

Primer 1 (AA2M2)

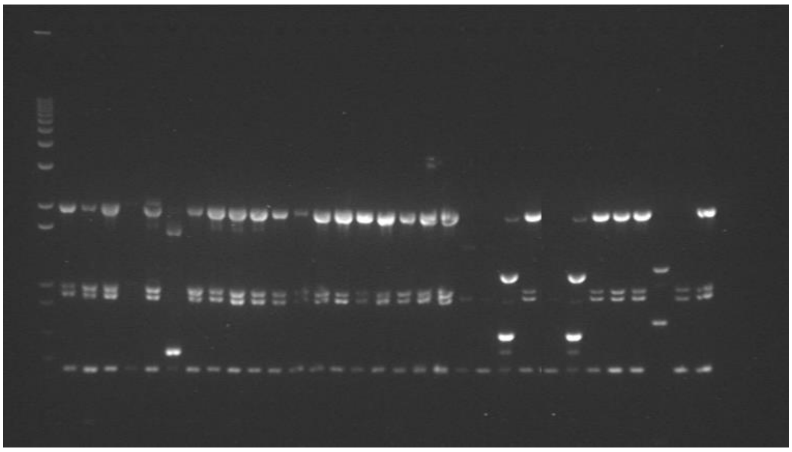

lane	Strain
1	Marker (100bp)
2	CO15 (internal control)
3	CO5
4	CO6
5	GE19
6	GE2
7	GE40
8	ITAB2
9	ITAC2
10	ITAE1
11	ITAE3
12	ITAG2
13	ITAH3
14	ITAI2
15	ITAJ1
16	ITAN1
17	ITAU1
18	ITAV1
19	LU2
20	LU3
21	C3
22	C5.scl
23	C5.19
24	CH1
25	Internal standard
23	NT3
24	Internal standard
25	CH16
26	CH7
27	PRC26
28	PT7
29	PT9
30	Negative control

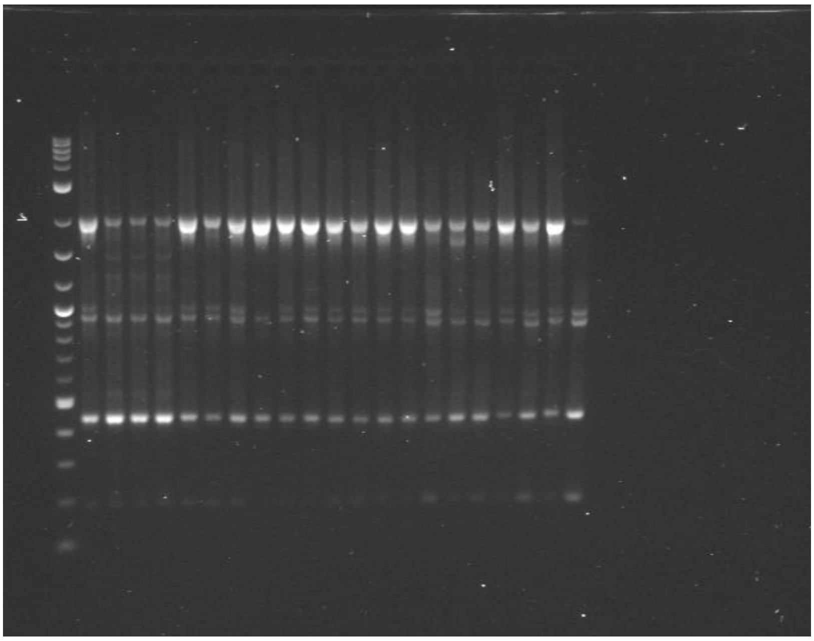

lane	strain
1	Marker (100bp)
2	CH14
3	CO1
4	Internal standard
5	Internal standard
6	Internal standard
7	Internal standard
8	Internal standard
9	Internal standard
10	Internal standard
11	Internal standard
12	Internal standard
13	Internal standard
14	Internal standard
15	M2A
16	Internal standard
17	Internal standard
18	Internal standard
19	NA5
20	NA6
21	NA8
22	CO5 (internal control)
23	Negative control

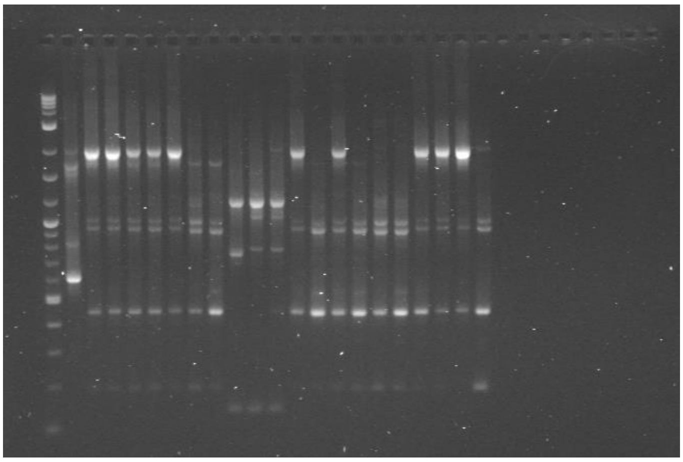

lane	strain
1	Marker (100bp)
2	Internal standard
3	Internal standard
4	Internal standard
5	Internal standard
6	Internal standard
7	Internal standard
8	NT1
9	NT2
10	PRC8
11	PRC21
12	PRC40
13	PT1
14	PT4
15	PT6
16	PT8
17	SPA1
18	SPA2
19	SPA3
20	SPA4
21	SPA5
22	CO5 (internal control)
23	Negative control

Primer 2 (AS15)

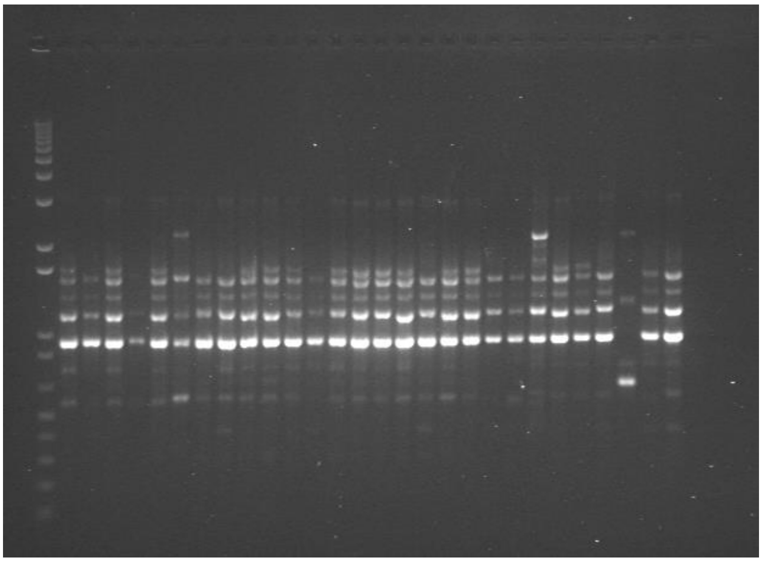

lane	strain
1	Marker (100bp)
2	CO15
3	CO5
4	CO6
5	GE19
6	GE2
7	GE40
8	ITAB2 (internal control)
9	ITAC2
10	ITAE1
11	ITAE3
12	ITAG2
13	ITAH3
14	ITAI2
15	ITAJ1 (internal control)
16	ITAN1
17	ITAU1
18	ITAV1
19	LU2
20	LU3
21	C3
22	C5 scl
23	C5.19
24	CH1
25	CH16 (internal control)
26	CH7
27	PRC26
28	PT7
29	PT9
30	Negative control

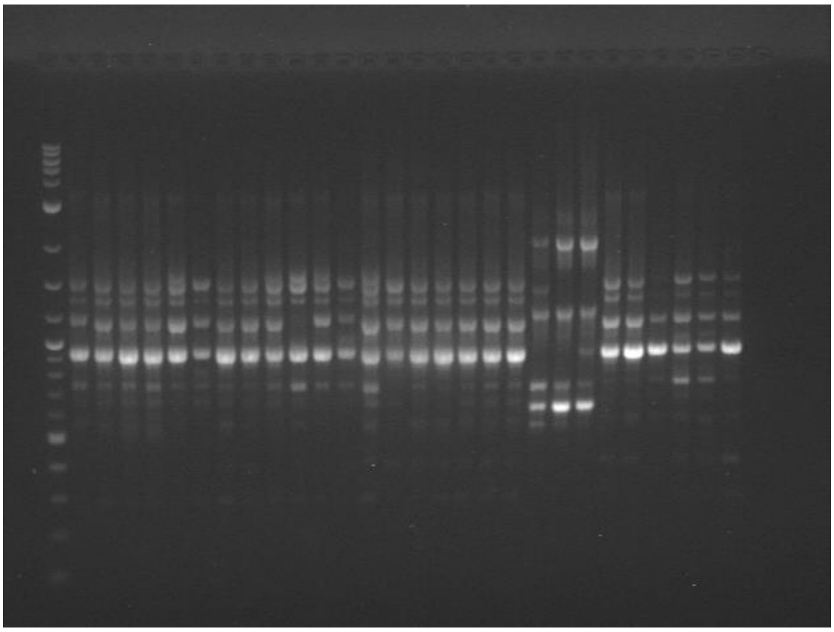

lane	strain
1	Marker (100bp)
2	CH14
3	CO1
4	Internal standard
5	Internal standard
6	Internal standard
7	Internal standard
8	Internal standard
9	Internal standard
10	Internal standard
11	Internal standard
12	MI02
13	Internal standard
14	Internal standard
15	NT1
16	NT2
17	PT1
18	PT4
19	PT6
20	PT8
21	PRC8
22	PRC21
23	PRC40
24	SPA1
25	SPA2
26	SPA3
27	SPA4
28	SPA5
29	CO5 (internal control)
30	Negative control

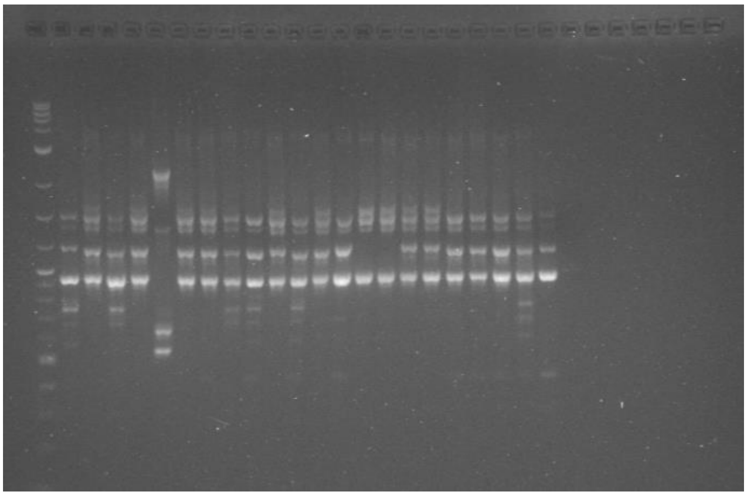

lane	strain
1	
2	Internal standard
3	NA5
4	Internal standard
5	Internal standard
6	NT3
7	Internal standard
8	Internal standard
9	Internal standard
10	Internal standard
11	Internal standard
12	Internal standard
13	Internal standard
14	Internal standard
15	Internal standard
16	Internal standard
17	Internal standard
18	Internal standard
19	SPA4A
20	SPA4B
21	SPA5A
22	SPA5B
23	CO5 (internal control)
24	Negative control

Primer 3 (AS15inv)

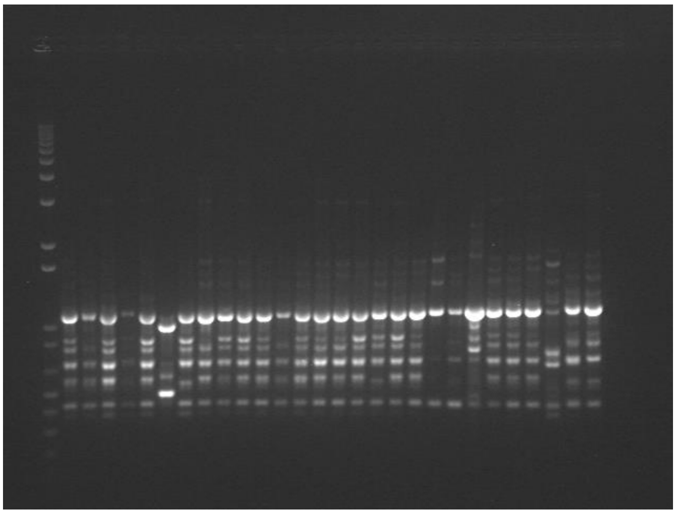

lane	strain
1	Marker (100bp)
2	CO15
3	CO5 (internal control)
4	CO6
5	GE19
6	GE2
7	GE40
8	ITAB2 (internal control)
9	ITAC2
10	ITAE1
11	ITAE3
12	ITAG2
13	ITAH3
14	ITAI2
15	ITAJ1 (internal control)
16	ITAN1
17	ITAU1
18	ITAV1
19	LU2
20	LU3
21	C3
22	C5 sc
23	C5.19
24	CH1
25	CH16 (internal control)
26	CH7
27	PRC26
28	PT7
29	PT9
30	Negative control

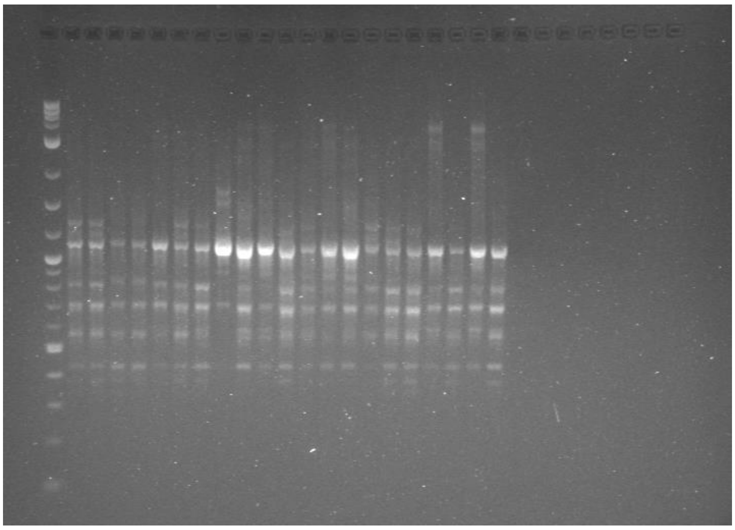

lane	strain
1	Marker (100bp)
2	CH14
3	CO1
4	Internal standard
5	Internal standard
6	Internal standard
7	Internal standard
8	Internal standard
9	Internal standard
10	Internal standard
11	Internal standard
12	Internal standard
13	Internal standard
14	Internal standard
15	M2A
16	NT3
17	Internal standard
18	Internal standard
19	NA5
20	Internal standard
21	Internal standard
22	CO5 (internal control)
23	Neg)ative control

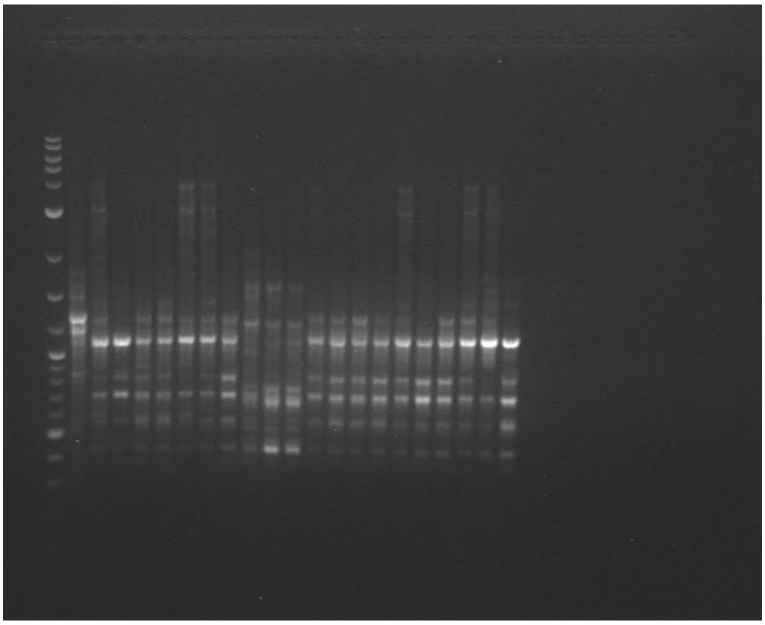

lane	strain
1	Marker (100bp)
2	Internal standard
3	Internal standard
4	Internal standard
5	Internal standard
6	Internal standard
7	Internal standard
8	NT1
9	NT2
10	PRC8
11	PRC21
12	PRC40
13	PT1
14	PT4
15	PT6
16	PT8
17	SPA1
18	SPA2
19	SPA3
20	SPA4
21	SPA5
22	CO5 (internal control)
23	Negative control

Primer 4 (AS4)

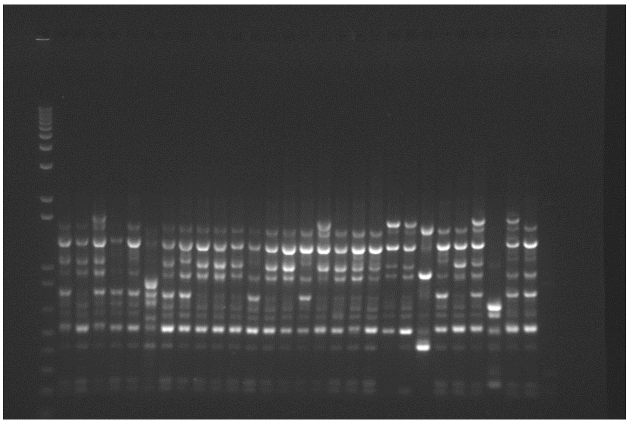

lane	strain
1	Marker (100bp)
2	CO15
3	CO5 (internal control)
4	CO6
5	GE19
6	GE2
7	GE40
8	ITAB2
9	ITAC2
10	ITAE1
11	ITAE3
12	ITAG2
13	ITAH3
14	ITAI2
15	ITAJ1
16	ITAN1
17	ITAU1
18	ITAV1
19	LU2
20	LU3
21	C3
22	C5 scl
23	NA16
24	CH1
25	CH16
26	CH7
27	PRC26
28	PT7
29	PT9
30	Negative control

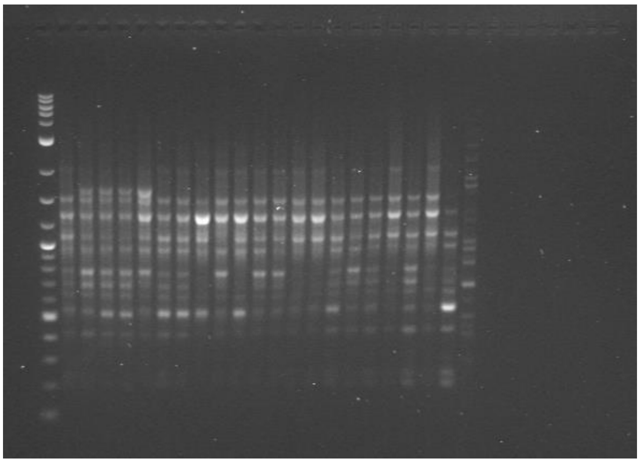

lane	strain
1	Marker (100bp)
2	CH14
3	CO1
4	Internal standard
5	Internal standard
6	Internal standard
7	Internal standard
8	Internal standard
9	Internal standard
10	Internal standard
11	Internal standard
12	Internal standard
13	Internal standard
14	Internal standard
15	M2A
16	Internal standard
17	Internal standard
18	Internal standard
19	NA5
20	Internal standard
21	NT3
22	CO5 (internal control)
23	Negative control

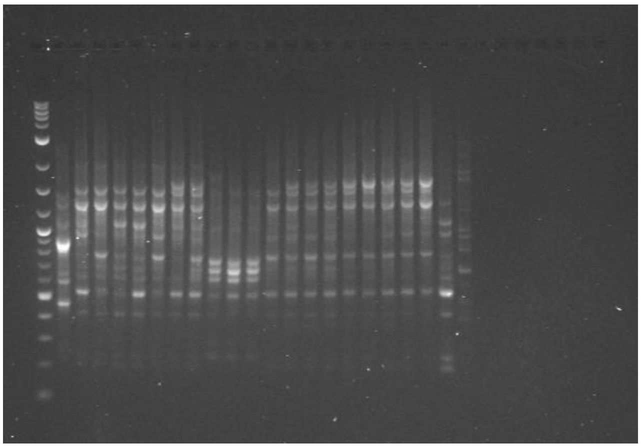

lane	strain
1	Marker (100bp)
2	Internal standard
3	Internal standard
4	Internal standard
5	Internal standard
6	Internal standard
7	Internal standard
8	NT1
9	NT2
10	PRC8
11	PRC21
12	PRC40
13	PT1
14	PT4
15	PT6
16	PT8
17	SPA1
18	SPA2
19	SPA3
20	SPA4
21	C5.19
22	CO5 (internal control)
23	Negative control

Primer 5 (Li5)

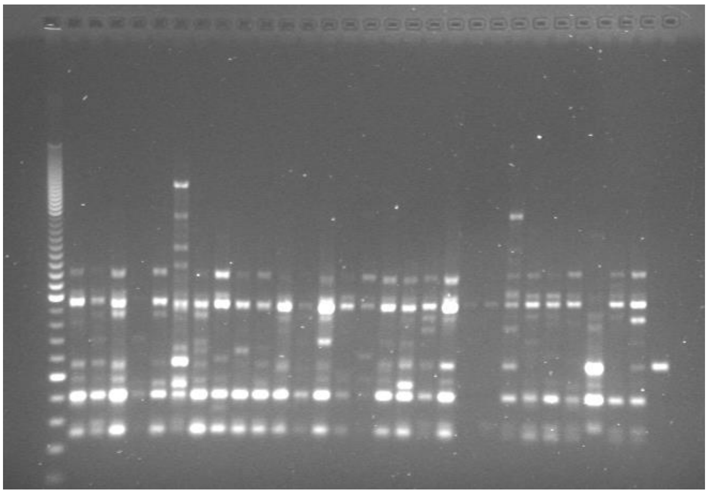

lane	strain
1	Marker (100bp)
2	CO15
3	CO5 (internal control)
4	CO6
5	GE19
6	GE2
7	GE40
8	ITAB2
9	ITAC2
10	ITAE1
11	ITAE3
12	ITAG2
13	ITAH3
14	ITAI2
15	ITAJ1
16	ITAN1
17	ITAU1
18	ITAV1
19	LU2
20	LU3
21	C3
22	C5 scl
23	C5.19
24	CH1
25	CH16
26	CH7
27	PRC26
28	PT7
29	PT9
30	Negative control

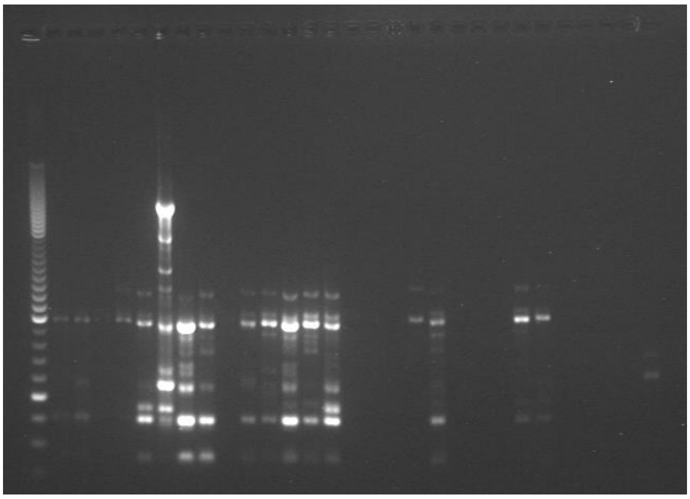

lane	strain
1	Marker (100bp)
2	CH14
3	CO1
4	Internal standard
5	Internal standard
6	Internal standard
7	Internal standard
8	Internal standard
9	Internal standard
10	Internal standard
11	Internal standard
12	Internal standard
13	Internal standard
14	Internal standard
15	M2A
16	Internal standard
17	Internal standard
18	Internal standard
19	NA5
20	Internal standard
21	Internal standard
22	CO5 (internal control)
23	Negative control

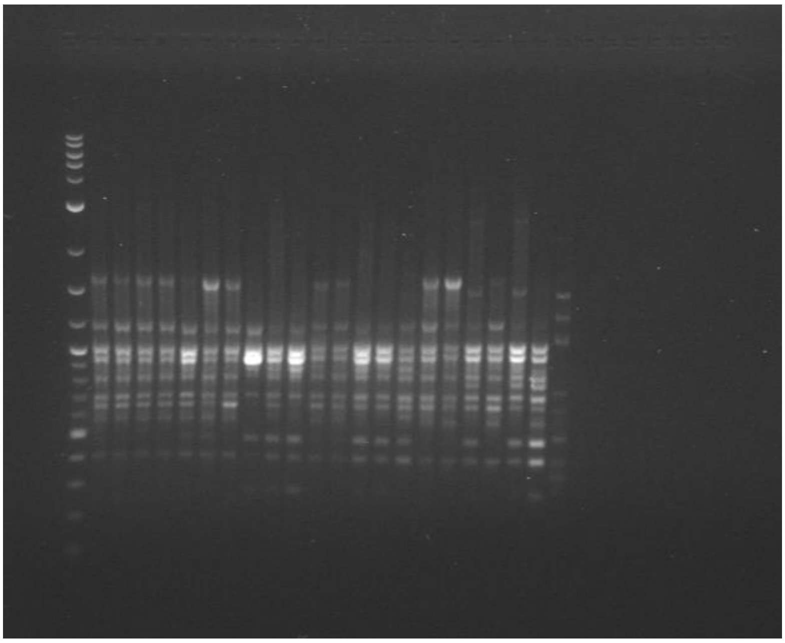

lane	strain
1	Marker (100bp)
2	Internal standard
3	NA5
4	Internal standard
5	CO1
6	Internal standard
7	MI2
8	NT1
9	NT2
10	PRC8
11	PRC21
12	PRC40
13	PT1
14	PT4
15	PT6
16	PT8
17	SPA1
18	SPA2
19	SPA3
20	SPA4
21	SPA5
22	CO5 (internal control)
23	Internal standard
24	Negative control

Primer 6 (L45)

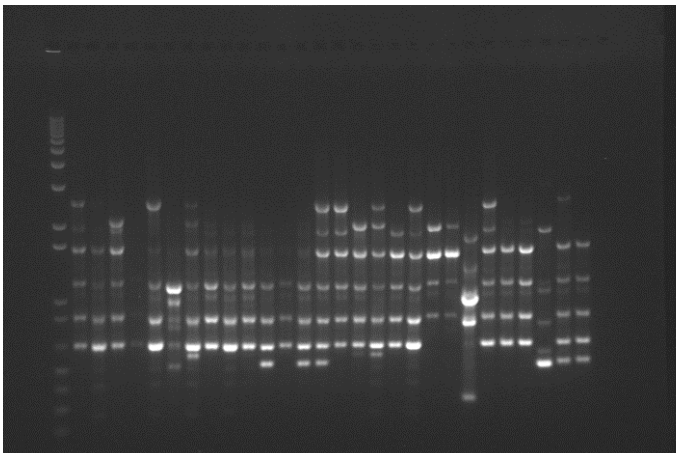

lane	strain
1	Marker (100bp)
2	CO15 (internal control)
3	CO5
4	CO6
5	GE19
6	NT3
7	GE40
8	ITAB2
9	ITAC2
10	ITAE1
11	ITAE3
12	ITAG2
13	ITAH3
14	ITAI2
15	ITAJ1
16	ITAN1
17	ITAU1
18	ITAV1
19	LU2
20	LU3
21	C3
22	C5 scl
23	C5.19
24	CH1
25	CH16
26	CH7
27	PRC26
28	PT7
29	PT9
30	Negative control

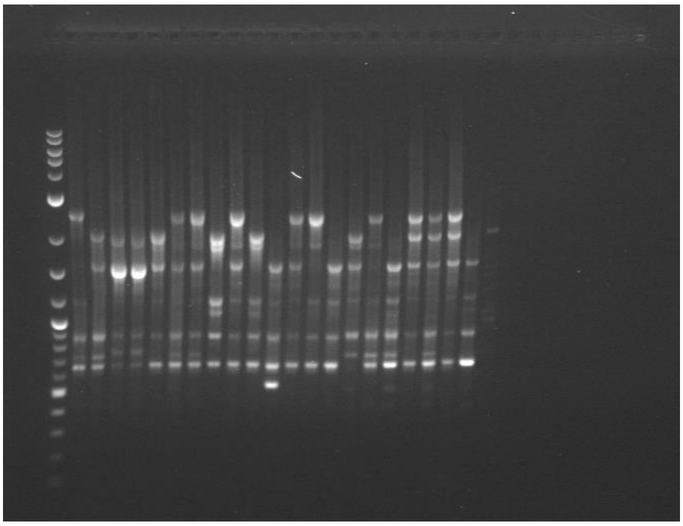

lane	strain
1	Marker (100bp)
2	CH14
3	CO1
4	Internal standard
5	Internal standard
6	Internal standard
7	Internal standard
8	Internal standard
9	Internal standard
10	Internal standard
11	Internal standard
12	Internal standard
13	GE2
14	Internal standard
15	M2A
16	Internal standard
17	Internal standard
18	Internal standard
19	NA5
20	Internal standard
21	PT9
22	CO5 (internal control)
23	Negative control

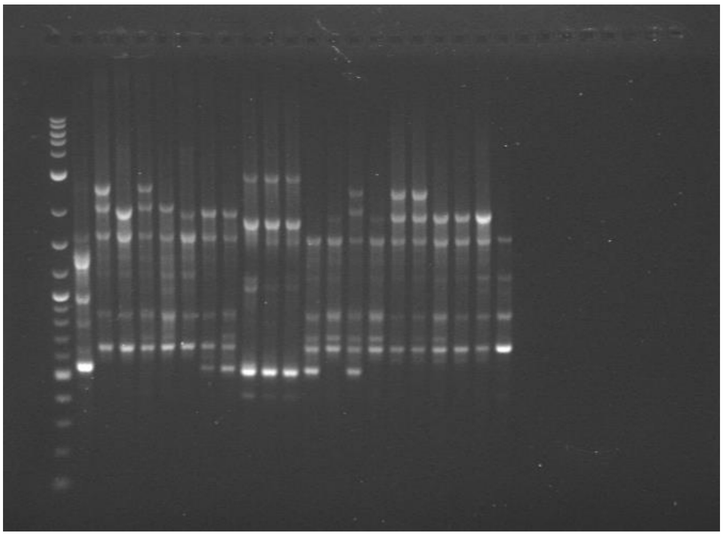

lane	strain
1	Marker (100bp)
2	Internal standard
3	Internal standard
4	Internal standard
5	Internal standard
6	Internal standard
7	Internal standard
8	NT1
9	NT2
10	PRC8
11	PRC21
12	PRC40
13	PT1
14	PT4
15	PT6
16	PT8
17	SPA1
18	SPA2
19	SPA3
20	SPA4
21	SPA5
22	CO5 (internal control)
23	Negative control
